# Divergent oncogenic signaling and immune microenvironment changes in low-grade serous ovarian cancer patients undergoing intraperitoneal chemotherapy

**DOI:** 10.1038/s41698-025-01182-3

**Published:** 2025-12-14

**Authors:** Isaac Bishara, Patrick A. Cosgrove, Sumana Majumdar, Colt A. Egelston, Oscar Colunga Flores, Brad Nakamura, Nora Ruel, Paul H. Frankel, Susan E. Yost, Sue Chang, Alexander Jung, Mihaela Cristea, Mustafa Raoof, Andrea H. Bild, Aritro Nath, Thanh H. Dellinger

**Affiliations:** 1 Department of Medical Oncology and Therapeutics Research, City of Hope National Medical Center, Duarte, CA USA; 2 Department of Immuno-Oncology, City of Hope National Medical Center, Duarte, CA USA; 3 Department of Surgery, City of Hope National Medical Center, Duarte, CA USA; 4 Department of Computation and Quantitative Medicine, City of Hope National Medical Center, Duarte, CA USA; 5 Department of Pathology, City of Hope National Medical Center, Duarte, CA USA; 6 Department of Diagnostic Radiology, City of Hope National Medical Center, Duarte, CA USA; 7 Regeneron Pharmaceuticals, New York, NY USA

**Keywords:** Cancer genomics, Ovarian cancer

## Abstract

Low-grade serous ovarian carcinoma (LGSOC) is a rare, treatment-resistant subtype of ovarian cancer characterized by peritoneal spread. Pressurized Intraperitoneal Aerosol Chemotherapy (PIPAC) delivers chemotherapy directly into the abdomen during serial laparoscopic surgeries, enabling repeated sampling of tumors for longitudinal analysis. Using single-nuclei RNA sequencing on 15 tumor samples across PIPAC cycles in two patients (a responder and a non-responder), we tracked molecular and cellular changes over time. Non-responder tumors showed upregulation of proliferation pathways (E2F, MYC, G2/M), KRAS signaling, epithelial-mesenchymal transition, and unfolded protein response, correlating with resistance. Responder tumors exhibited downregulation of proliferation and stress pathways but activated alternative survival mechanisms (PI3K, Wnt/β-catenin, Notch). Archetype analysis revealed dynamic shifts in metabolic and immune-related subpopulations in the responder tumors, while immunoprofiling showed greater immunosuppression in non-responder tumors. Despite the small cohort, these exploratory observations highlight tumor adaptation and underscore the need for multi-targeted therapies addressing proliferation, stress, survival, and immune evasion.

## Introduction

Low-grade serous ovarian carcinoma (LGSOC) is an uncommon subtype of epithelial ovarian cancer (EOC), accounting for approximately 5–10% of all EOC cases^1^. Despite its indolent nature, approximately 70% of patients with advanced-stage LGSOC experience disease recurrence^2^. LGSOC poses significant clinical challenges due to its inherent resistance to conventional therapies. In contrast to the more prevalent high-grade serous ovarian carcinoma (HGSOC), patients diagnosed with LGSOC typically present at a younger age and demonstrate significantly lower responsiveness to systemic chemotherapy, with response rates below 5% in recurrent disease settings^3^.

LGSOC is characterized by alterations in the MAPK/RAS and PI3K-AKT signaling pathways, contributing to therapeutic resistance and disease progression^4,5^. Managing recurrent LGSOC is challenging due to extensive, unresectable peritoneal metastases that respond poorly to systemic treatments. Localized treatments, such as intraperitoneal (IP) chemotherapy, have demonstrated efficacy but have not been comprehensively evaluated in LGSOC patients^6,7^.

Pressurized Intraperitoneal Aerosol Chemotherapy (PIPAC) is a novel treatment that improves chemotherapy delivery to peritoneal metastases^8–10^. PIPAC involves aerosolizing chemotherapy into microdroplets using a micropump and high-pressure injector during laparoscopic surgery^11^. The transiently elevated intra-abdominal pressure generated by CO2 gas helps overcome interstitial pressure within tumors and enables improved drug diffusion into tumors. Additionally, the aerosolized drug’s superior distribution enables better penetration around fibrosis and loculations, potentially accessing areas unreachable by traditional intraperitoneal chemotherapy.

To better understand therapeutic resistance mechanisms in LGSOC and identify potential strategies to overcome chemoresistance, we conducted the first longitudinal analysis of single-nuclei transcriptomic changes occurring through successive chemotherapy cycles. To do this, we analyzed tumor samples from two heavily pretreated LGSOC patients undergoing PIPAC therapy, who each represented a treatment response and treatment failure, to PIPAC, respectively. Using single-nuclei RNA sequencing (snRNA-seq), we captured molecular snapshots of the evolving tumor microenvironment by analyzing spatial and sequentially collected tumor samples prior to each chemotherapy treatment cycle to identify cellular pathways linked to therapeutic response over time. Our preliminary findings provide a foundation for larger-scale studies to refine molecular-based treatment strategies by highlighting essential pathways, tumor microenvironment interactions, and immune-mediated mechanisms that significantly influence therapeutic outcomes and chemoresistance in LGSOC patients.

## Results

### Clinical profile and efficacy of PIPAC in recurrent LGSOC

**Fig. 1:**
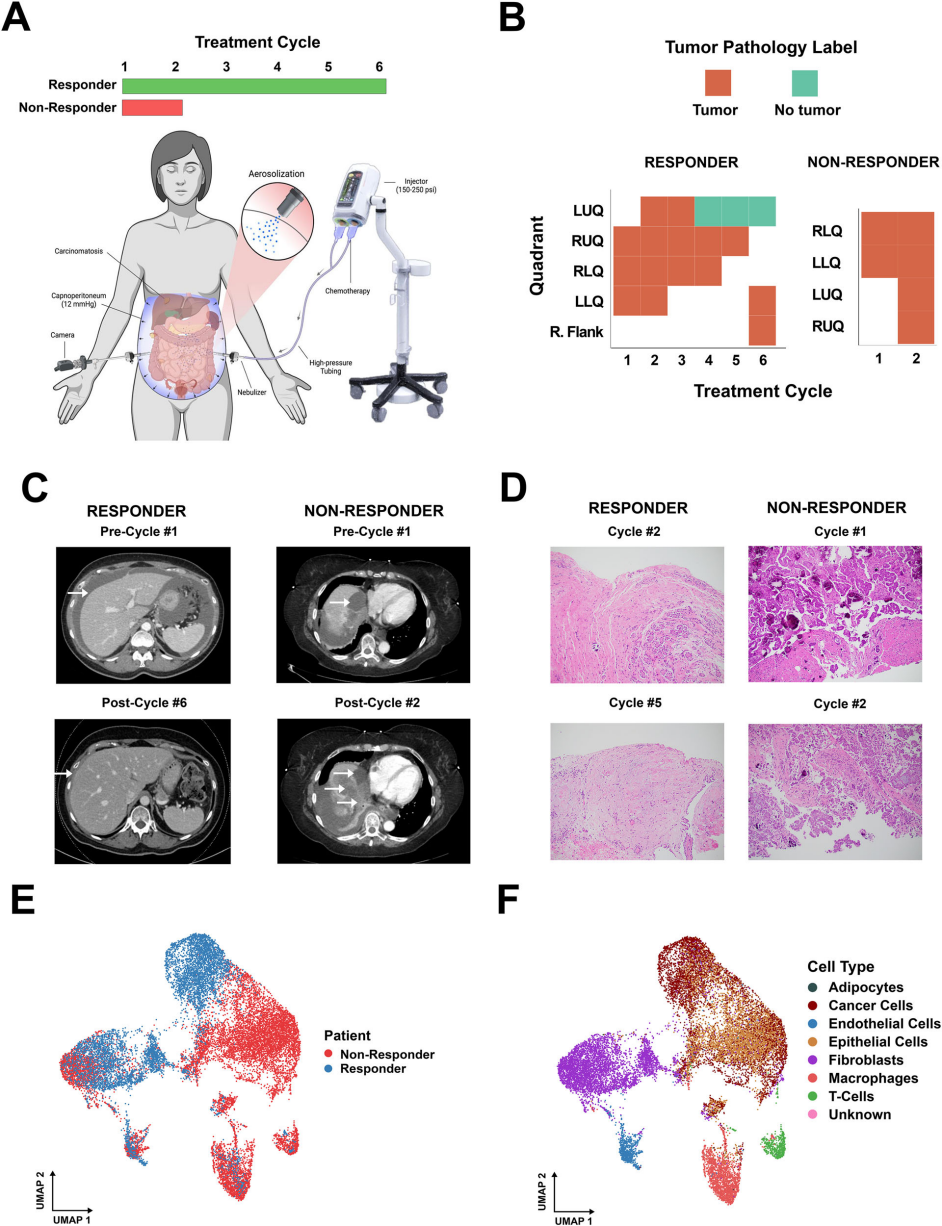
Study overview, patient imaging, pathology, and single-nuclei profiling. **A** Scheme of the pressurized intraperitoneal aerosol chemotherapy (PIPAC) procedure and treatment course for the responder (green) and non-responder (red) patients. Chemotherapy is aerosolized by a nebulizer through a high-pressure injector during laparoscopic surgery under CO₂ capnoperitoneum, allowing better drug distribution and tissue penetration. The responder received six PIPAC cycles; the non-responder discontinued due to progression of disease after two cycles. Created with Biorender.com. **B** Histologic assessments of peritoneal tumor biopsies taken from multiple abdominal quadrants across treatment cycles. Each colored tile represents whether pathology was positive (tumor present), negative (no tumor), for a given quadrant and cycle. **C** Contrast-enhanced computed tomography (CT) scans show the clinical response in the responder (left) and the non-responder (right). The baseline CT scans, taken before cycle #1, and the post-treatment CT scans, after cycle #6 for the responder and after cycle #2 for the non-responder, show resolved ascites in the responder (left) compared to progressive hepatic metastasis in the non-responder (right). **D** Representative hematoxylin and eosin (H&E) staining of tumor samples obtained at various PIPAC cycles. The responder’s biopsies (left panels) show decreased tumor cells and increasing fibrosis by cycle #5, while the non-responder’s samples (right panels) reveal persistent viable tumor at cycles #1 and #2. **E** Uniform Manifold Approximation and Projection (UMAP) plot of single-nuclei transcriptomic data shows the responder (blue) and non-responder (red). **F** The UMAP visualization shows the same single-nuclei dataset as in (**F**), but now colored by assigned cell types: it displays cancer cells, fibroblasts, and T-cells contributing to the tumor microenvironment in each patient sample.

We present two patients with recurrent, late-stage LGSOC who were extensively pretreated with multiple chemotherapy lines (Table 1, Supplementary Document S1). Peritoneal metastasis samples were collected during every cycle of PIPAC (Fig. 1A, Supplementary Table S1). All samples were obtained under protocols approved by the Institutional Review Board (IRB) # 19184 of City of Hope, under informed consent before collection. Both patients received aerosolized intraperitoneal cisplatin (10.5 mg/m^2^) and doxorubicin (2.1 mg/m^2^) every six weeks, with the intent to undergo 3 or more PIPAC cycles. Tissues from multiple abdominal quadrants were sampled during the laparoscopic surgery before and after each PIPAC cycle, and samples were examined for tumor cells by a certified pathologist (Fig. 1B). A total of 21 biopsies were collected. After quality control to select high-quality cells from cancer-containing samples, we analyzed 10 biopsies from the responder and 5 biopsies from the non-responder. The first patient (“non-responder”) was a 69-year-old female with a history of platinum-resistant LGSOC and ten prior lines of treatment, who demonstrated extraperitoneal progression of disease after 2 PIPAC cycles of therapy. Molecularly, this patient’s tumor harbored an NRAS mutation, consistent with the MAPK-mutant LGSOC subtype that has been associated with micropapillary stromal invasion and poor survival based on a recent whole-exome sequencing analysis identifying molecular subcohorts (Supplementary Document S1)^12^. Following progression of disease, the patient withdrew from the study after 2 PIPAC cycles. The second patient (“responder”) was a 59-year-old female with a history of platinum-resistant LGSOC with 6 prior lines of therapy who received a total of 6 PIPAC cycles with excellent response, as demonstrated by a partial response by imaging, substantial tumor marker (CA-125) level reduction, resolution of moderate ascites, and histologic tumor regression of the peritoneal tumors sampled.

**Table 1:** Patient information

	Responder	Non-responder
**Age at diagnosis**	59	68
**Race/Ethnicity**	Non-Hispanic White	Non-Hispanic White
**Body mass index at cycle 1**	24.4	32.9
**ECOG performance status**	1	1
**Prior lines of therapy**	6	10
**Baseline metastatic status**	Intraperitoneal only	Extraperitoneal and intraperitoneal
**PIPAC cycles received**	6	2
**Baseline PCI**	20	20
**Cycle 2 PCI**	19	14
**Baseline mean PRGS**	3	3.5
**Baseline ascites volume**	1500 mL	10 mL
**Best response per RECIST**	Partial response	Progressive disease
**PFS (months)**	21.6	1.7
**OS (months from PIPAC initiation)**	33.6	11.9
**OS months from initial diagnosis**	124.3	92.9
**Off-treatment reason**	Treatment completed per protocol	RECIST-based progression
**Progression type**	Intraperitoneal	Extraperitoneal and liver parenchymal
**Grade**	Low grade	Low grade
**Stage**	3	4

This table shows the clinical annotations for the two patients in the study.

*ECOG* Eastern Cooperative Group, *PIPAC* pressurized intraperitoneal aerosolized chemotherapy, *PCI* peritoneal carcinomatosis index, *PRGS* peritoneal regression grading score, *RECIST* response evaluation criteria in solid tumors, *PFS* progression-free survival, *OS* overall survival.

Contrast-enhanced computed tomography scans (CT) were performed to determine the response. The responder’s CT imaging after cycle 3 demonstrated the resolution of ascites (Fig. 1C, left), while the non-responder’s imaging showed the rapid growth of a liver lesion seen on CT imaging between cycles 1 and 2, characterized by an increase in size of the heterogeneous lobulated mass arising from the medial dome of the liver (Fig. 1C, right).

Histologically, the responder’s tumor demonstrated decreased tumor cells and increasing fibrosis by hematoxylin and eosin (H&E) staining of tumor samples by cycle 5 (Fig. 1D, left), as compared to the non-responder’s persistent viable tumor staining at cycle 2 (Fig. 1D, right).

To investigate the molecular basis underlying these differential responses, snRNA-sequencing was performed on a representative subset of the 21 collected samples. After quality control to select only high-quality cells and filtering for cancer-containing samples collected from tumor sites prior to each PIPAC cycle, we retained 18,351 cells for downstream analysis, including 6776 cells derived from 10 biopsies of the responder patient’s tumor and 11,575 cells from 5 biopsies of the non-responder patient (Fig. 1E). Besides malignant cells, cell-type annotation showed adipocytes, endothelial cells, epithelial cells, fibroblasts, macrophages, and T-cell lymphocytes (Fig. 1F, Supplementary Fig. S1A). However, the average number of immune cells detected in the responder’s tumor, including T-cells (5.33 cells/sample) (Supplementary Fig. S1B**)** and macrophages (23.5 cells/sample) (Supplementary Fig. S1C**)**, was too low to facilitate a comprehensive transcriptional tumor microenvironment (TME) analysis.

### Pathway activity alterations in response to PIPAC treatment

**Fig. 2:**
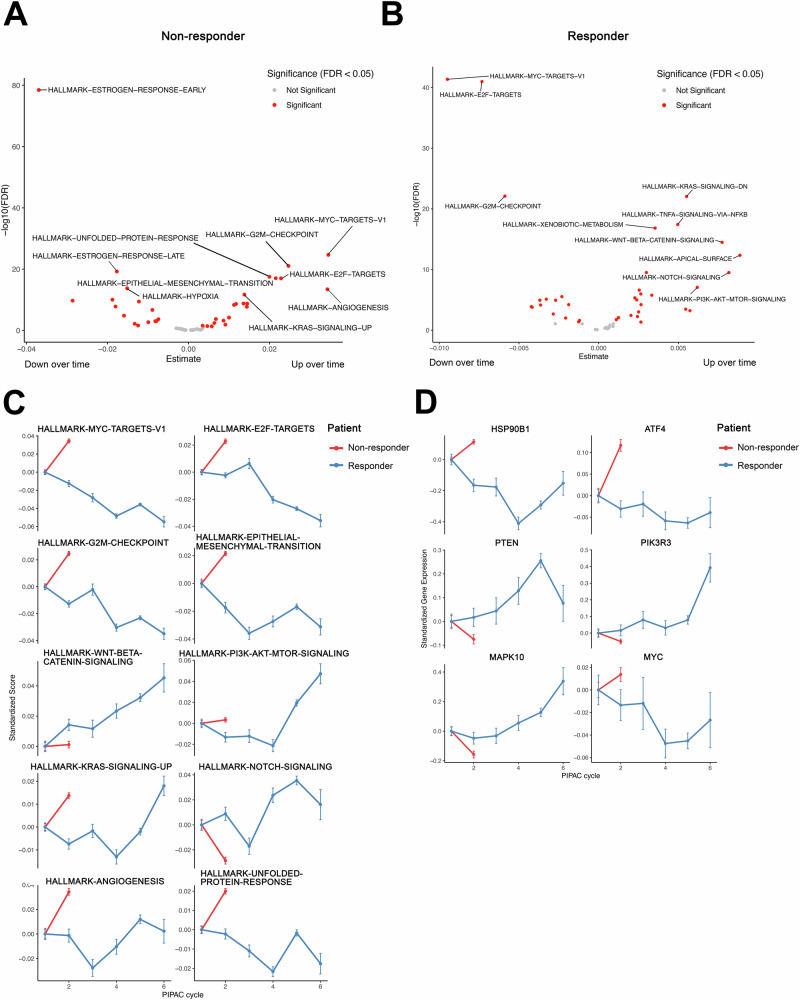
Pathway activity alterations in response to PIPAC treatment. **A**, **B** Volcano plots displaying temporal pathway activity changes identified through linear modeling of ssGSEA scores across all treatment cycles. **A** The non-responder tumors show significant upregulation of proliferation-associated pathways (E2F targets, G2/M checkpoint, MYC targets) and KRAS signaling, along with increased activity in EMT and UPR. Estrogen response pathway activity is significantly reduced. FDR < 0.05 was considered significant. **B** In contrast, the responder tumors exhibit a significant decrease in proliferation-associated pathways (E2F targets, G2/M checkpoint, MYC targets) and KRAS signaling while showing increased PI3K, Wnt/beta-catenin, and Notch signaling. FDR < 0.05 was considered significant. **C** Line plots showing divergent temporal changes in selected hallmark pathways across PIPAC cycles in both patients. The non-responder tumors (red) exhibit increasing proliferation and stress-related pathways over time, whereas the responder tumors (blue) show decreasing activity in proliferation pathways and EMT while increasing alternative survival pathways such as PI3K signaling. **D** Temporal changes in the expression of key genes associated with UPR (HSP90B1, ATF4), tumor suppression (PTEN), PI3K signaling (PIK3R3), and stress adaptation (MAPK10, MYC). In the non-responder tumors, HSP90B1 and ATF4 increase, reflecting heightened stress adaptation. In contrast, the responder tumors exhibit upregulation of PTEN and MAPK10 while MYC levels decline, supporting a shift away from proliferative signaling.

To investigate the dynamics of key cancer pathways during PIPAC treatment in both patients, we applied a linear model to the single-sample Gene Set Enrichment Analysis (ssGSEA) Hallmark pathway scores across all treatment cycles. Our analysis of the non-responder revealed a pronounced rise in proliferation pathways, including E2F targets (estimate = 0.022, FDR = 1.0 × 10^-17^) and the G2/M checkpoint (estimate = 0.024, FDR = 8.7 × 10^-22^), over the two PIPAC cycles (Fig. 2A, Supplementary Table S2). This increase was further accompanied by upregulation of MYC targets V1 pathway (estimate = 0.034, FDR = 2.1 × 10^-25^), indicating accelerated tumor growth and an inadequate therapeutic response. Notably, among the most prominently upregulated MYC-regulated genes were those involved in protein synthesis and gene regulation, including RPLP0 (estimate = 0.221, FDR = 9.8 × 10^-11^), RPL18 (estimate = 0.165, FDR = 1.8 × 10^-06^), RPS2 (estimate = 0.231, FDR = 2.7 × 10^-08^), RPS5 (estimate = 0.146, FDR = 2.0 × 10^-06^), and HNRNPU (estimate = 0.196, FDR = 1.8 × 10^-10^), HNRNPC (estimate = 0.243, FDR = 1.4 × 10^-12^), and HNRNPA2B1 (estimate = 0.174, FDR = 9.7 × 10^-09^) (Supplementary Fig. S2A, Supplementary Table S3). In addition to proliferation, we also observed an increase in the KRAS signaling pathway in this patient (estimate = 0.013, FDR = 1.9 × 10^-12^) (Fig. 2A**)**. In addition, epithelial-mesenchymal transition (EMT) (estimate = 0.021, FDR = 9.1 × 10^-18^) and angiogenesis (estimate = 0.034, FDR = 3.7 × 10^-14^) pathways were significantly elevated. We also detected higher activity in the unfolded protein response (UPR) pathway (estimate = 0.019, FDR = 3.1 × 10^-18^), likely reflecting an adaptive mechanism to therapeutic stress. By applying the temporal linear modeling to individual genes, we identified upregulated UPR-related genes, including HSP90B1 (estimate = 0.114, FDR = 3.0 × 10^-05^) and the classic UPR factor ATF4 (estimate = 0.116, FDR = 1.0 × 10^-06^) (Supplementary Fig. S2B). Altogether, these alterations suggest that the tumor is shifting to a more aggressive, growth-focused phenotype under PIPAC therapy. Concurrently, a notable decrease in the estrogen response pathway (estimate = -0.036, FDR = 3.3 × 10^-79^) points to reduced hormonally governed growth restraint.

In contrast, the responder's tumors exhibited a decrease in proliferation pathways over six PIPAC cycles (Fig. 2B, C). E2F targets (estimate = −0.007, FDR = 1.0 × 10^-41^), MYC targets (estimate = −0.009, FDR = 4.2 × 10^-42^), and the G2/M checkpoint (estimate = −0.005, FDR = 7.6 × 10^-23^) all showed significant reduction. Several key genes within the MYC pathway involved in protein synthesis and gene regulation, including RPL6 (estimate = −0.081, FDR = 6.4 × 10^-19^), RPL14 (estimate = −0.065, FDR = 1.2 × 10^-20^), RPL34 (estimate = −0.071, FDR = 1.2 × 10^-13^), RPL22 (estimate = −0.034, FDR = 6.1 × 10^-06^), RPL18 (estimate = −0.039, FDR = 2.3 × 10^-06^), RPS2 (estimate = −0.079, FDR = 6.6 × 10^-18^), RPS6 (estimate = −0.054, FDR = 5.0 × 10^-11^), RPS3 (estimate = −0.048, FDR = 2.8 × 10^-06^), RPS10 (estimate = −0.039, FDR = 8.2 × 10^-11^), HNRNPU (estimate = −0.066, FDR = 2.3 × 10^-09^), HNRNPA3 (estimate = −0.063, FDR = 6.3 × 10^-11^), and HNRNPA2B1 (estimate = −0.085, FDR = 4.2 × 10^-15^), were significantly downregulated (Supplementary Fig. S2D, Supplementary Table S3). This finding aligns with the observed reduction in protein secretion pathway activity. Meanwhile, EMT, typically linked with cancer cell survival and resistance, was significantly reduced (estimate = −0.003, FDR = 1.1 × 10-5). We further noted increased activity in alternative growth and survival pathways, including PI3K (estimate = 0.006, FDR = 8.6 × 10^-08^), Wnt/beta-catenin (estimate = 0.007, FDR = 3.2 × 10^-15^), Notch signaling (estimate = 0.008, FDR = 3.0 × 10^-10^), and KRAS signaling (estimate = 0.0003, FDR = 0.49). Given the critical role of the PI3K pathway in LGSOC, we performed an in-depth analysis of its member genes (Supplementary Fig. S2F vs Supplementary Fig. S2C). While HRAS expression was significantly reduced (estimate = −0.035, FDR = 8.2 × 10^-11^) and PTEN was upregulated (estimate = 0.058, FDR = 1.5 × 10^-07^), PIK3R3 (estimate = 0.028, FDR = 6.3 × 10^-3^), JNK-1 (MAPK8) (estimate = 0.045, FDR = 7.3 × 10^-05^), and JNK-3 (MAPK10) (estimate = 0.043, FDR = 1.4 × 10^-4^) were all significantly increased. In addition, augmented TNFA signaling (estimate = 0.004, FDR = 3.7 × 10^-18^) may signify diminished reliance on proliferation and a compensatory shift toward alternative survival mechanisms, as cancer cells adapt to treatment stress. In contrast to the non-responder’s tumors, HSP90B1 (estimate = −0.064, FDR = 4.7 × 10^-10^) and ATF4 (estimate = −0.013, FDR = 0.01) stress genes showed significant downregulation in the reponder’s tumors (Fig. 2D, Supplementary Fig. S2E). Altogether, these findings highlight the distinct and dynamic reprogramming of proliferative, survival, and stress-related pathways under PIPAC therapy in the non-responder and responder, elucidating how tumors may adapt or succumb to therapeutic pressure.

### Identification and characterization of cellular archetypes across treatment cycles

**Fig. 3:**
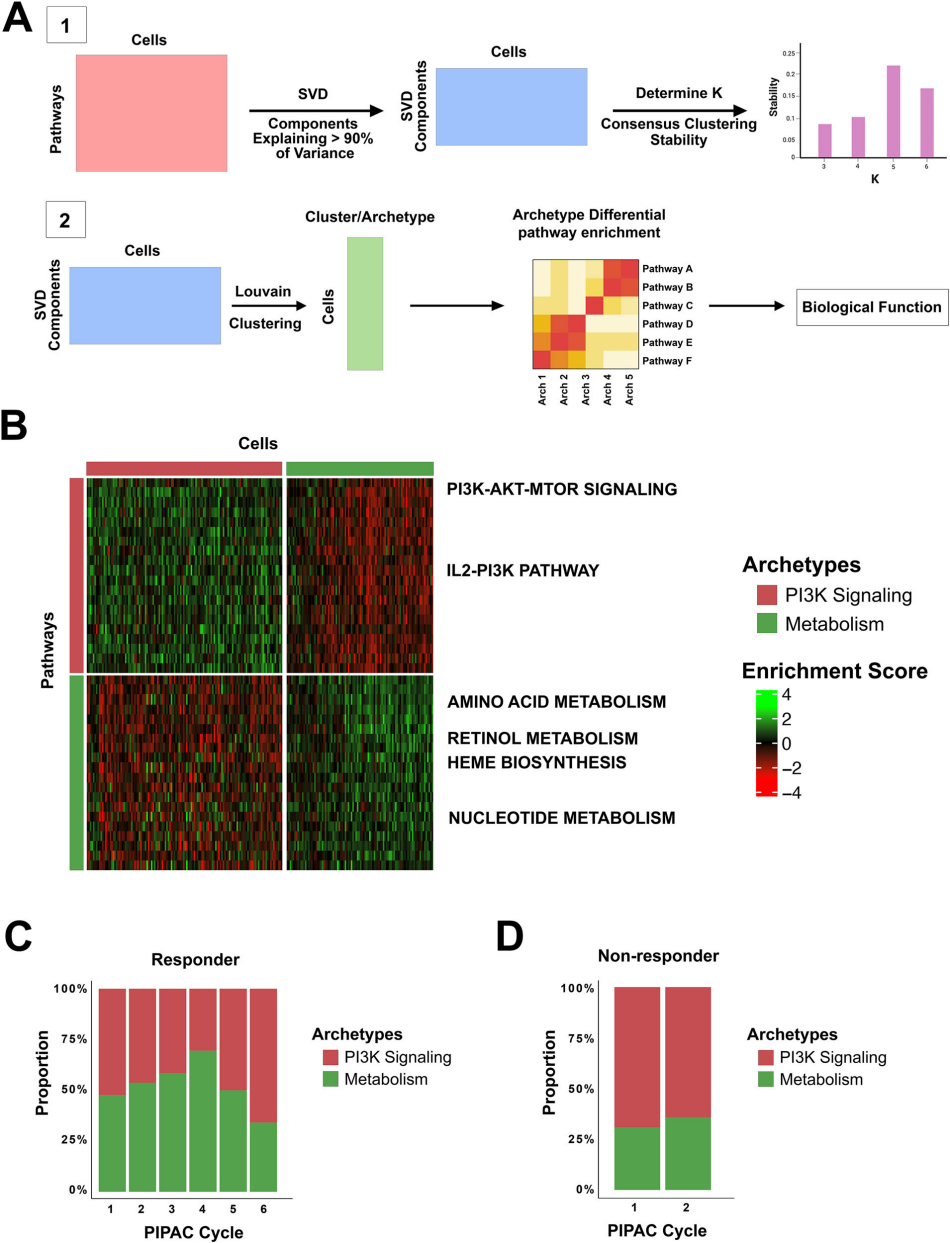
Identification and characterization of cancer cell archetypes across treatment cycles. **A** Schematic representation of the analytical workflow used to identify cancer cell archetypes. ssGSEA derived from the hallmark gene set was reduced via singular value decomposition (SVD), retaining components explaining 90% of the variance. The optimal number of archetypes (K) was determined using a consensus clustering stability algorithm, and cells were subsequently clustered into archetypes using the Louvain algorithm (Seurat implementation). DPE analysis was then performed to define the biological function of each archetype based on the top 20 enriched pathways using the Wilcoxon Rank Sum test. **B** Heatmap illustrating pathway enrichment scores distinguishing the two primary archetypes identified across patients. Archetype 1 (PI3K signaling) shows enrichment of PI3K-related pathways and immune signaling, while Archetype 2 (Metabolism) shows enrichment of metabolic pathways, including amino acid metabolism, heme metabolism, nucleoside metabolism, and retinoic acid metabolism. **C** Proportion of cells assigned to each archetype over six treatment cycles in the responder patient, demonstrating dynamic shifts in archetype composition, with the metabolism archetype initially increasing before rapidly declining as the PI3K signaling archetype expanded to dominate. **D** Proportion of cells assigned to each archetype over two treatment cycles in the non-responder patient, demonstrating stable dominance of the PI3K signaling archetype.

Building on these pathway-level observations, we next sought to characterize the underlying cellular heterogeneity driving these responses by identifying and defining distinct cellular archetypes across treatment cycles. We applied singular value decomposition (SVD) to the single-cell pathway ssGSEA scores derived from Hallmark, Kyoto Encyclopedia of Genes and Genomes (KEGG), Reactome, Biocarta, and Pathway Interaction Database (PID) gene set databases (Fig. 3A). Given that samples from each patient were processed separately, we excluded the top SVD component associated with the observed batch effect from the downstream analysis (Supplementary Fig. S3A–D) (see “Methods” section). The consensus clustering stability algorithm, used to determine the optimal number of archetypes (K), resulted in two archetypes used for clustering the individual cells (Supplementary Fig. S3E, F). To interpret each archetype’s biological function, we performed a Wilcoxon Rank Sum test to compare the two clusters/archetypes, using the top 20 enriched pathways to define their functional identities (Fig. 3B, Supplementary Fig. S4A). We found the first archetype to be enriched in PI3K signaling along with immune pathways (T-cell and B-cell receptor signaling), while the second archetype was enriched in several metabolic pathways, including amino acid metabolism, heme metabolism, nucleoside transport and metabolism, and retinoic acid metabolism. Studying the proportions of cells belonging to each archetype over treatment cycles revealed that the responder patient showed a dynamic non-linear progression (Fig. 3C). The cancer cell population was almost equally divided across the two archetypes. However, over 4 treatment cycles, the cancer cells associated with the metabolism archetype grew to constitute 69.5% of the population at cycle 4. Interestingly, this population decreased to 34.1% of the total population at cycle 6 as the PI3K/immune-enriched population became the majority. In contrast, PI3K signaling was enriched in 69.3 of the cancer cells in the non-responder’s tumors prior to cycle 1, and decreased to 64.3% after cycle 2, while the remaining cells were enriched in the metabolism archetype (Fig. 3D). Over the two sampled timepoints, there was no notable change in these proportions.

To confirm these findings, we expanded the same analysis pipeline to individual patients. Two archetypes were identified in the responder **(**Supplementary Fig. S5A, B). The first archetype was involved in the same metabolism pathways previously identified, while the second archetype was characterized by receptor tyrosine kinase (RTK) signaling and immune pathways, including T-cell receptor signaling, interleukin and cytokine signaling, and innate and adaptive immune response (Supplementary Fig. S5c). Temporally, the metabolism archetype showed the same trend as before, where it grew from 46.9% (cycle 1) to 69.1% (cycle 4), then declined to 34.1% (cycle 6) as the RTK/immune archetype recovered and constituted the majority (65.9%) of cells by the 6th cycle (Supplementary Fig. S5D).

In the non-responder, three archetypes were identified and were associated with metabolism, RTK/immune (as described previously), and an additional archetype representing protein translation and ribosomes (Supplementary Fig. S6A–C). The strongest change over the two treatment cycles was associated with a depletion of the RTK/immune archetype, which decreased from 45.9% to 25.2% (Supplementary Fig. S6D). This was followed by the translation archetype, which expanded from 19.8% to 31.8% while the metabolism archetype showed a modest increase from 34.3% to 43%.

Given the non-linear nature of the temporal dynamics in the responder patient’s tumor, we briefly investigated the genetic subclonal evolution of malignant cells over the six treatment cycles. Using the inferred copy number alteration profile, we clustered the malignant cells into two clusters using the K-means algorithm (K = 2). The relative abundance of the cell clusters across treatment cycles mirrored the abundance of the PI3K and Metabolism archetypes to a great extent (Supplementary Fig. S7). These results suggest a simultaneous non-linear selection process of both the genotype and phenotype under treatment.

### Differential immune microenvironment changes induced by PIPAC in LGSOC

Given the enrichment and depletion of the immune archetypes in responder and non-responder, respectively, we evaluated the immune cell composition of the tumor microenvironment (TME) in both patients’ tumors and tracked how it evolved under PIPAC. Due to the low number of detected immune cells, potentially due to sampling quality and platform-specific differences (see “Methods” section), particularly macrophages and T-cells in the responder, a snRNA-based immune analysis was not possible. Instead, we performed immunostaining on tissue slides to quantify different immune cell populations.

Immunostaining revealed no significant disparity between the total CD4+ or CD8+ T-cell populations in the responder versus the non-responder (Supplementary Fig. S8B, C).

**Fig. 4:**
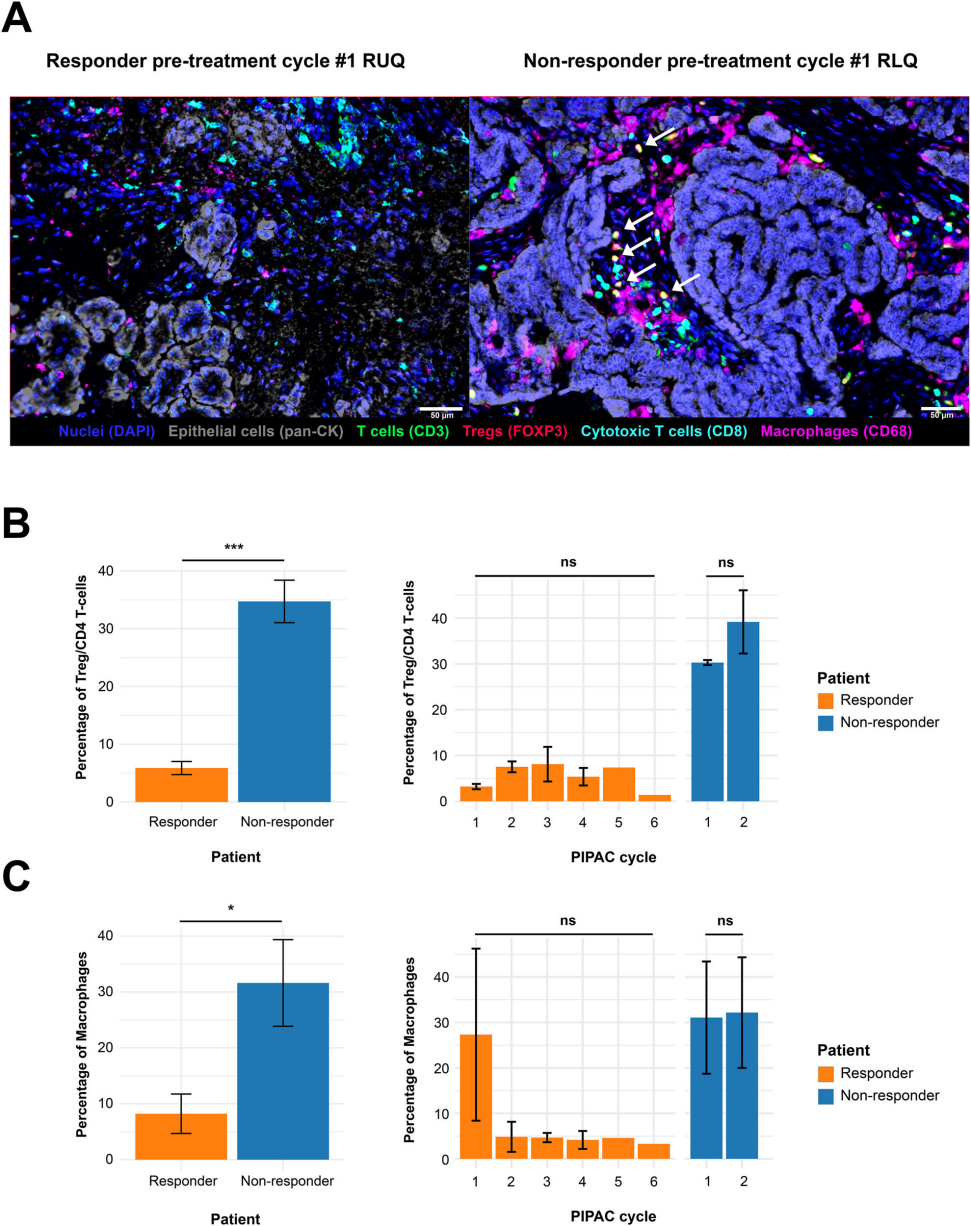
Immunostaining analysis reveals distinct immune cell compositions in responder and non-responder patients. **A** Representative multiplex immunofluorescence images of tumor samples from responder (left) and non-responder (right) patients at pre-treatment cycle #1. Stains include DAPI (nuclei, blue), pan-cytokeratin (epithelial cells), CD3 (T-cells, green), FOXP3 (regulatory T-cells, red), CD8 (cytotoxic T-cells, cyan), and CD68 (macrophages, magenta). White arrows indicate Tregs in the non-responder sample. Scale bars = 50 µm. **B** Quantification of Tregs as a percentage of CD4+ T-cells. Left panel: Average Treg proportions across all timepoints, showing significantly higher Treg frequencies in the non-responder patient. Right panel: Temporal dynamics of Treg proportions over PIPAC cycles, demonstrating stable low levels in the responder and a sharp increase in the non-responder over two cycles. **C** Quantification of macrophages. Left panel: Average macrophage proportions across all timepoints, indicating a higher macrophage burden in the non-responder patient. Right panel: Temporal dynamics over PIPAC cycles reveal an initial decline in macrophages in the responder, whereas macrophage levels remain consistently high in the non-responder. Statistical analysis was performed using unpaired two-tailed *t*-tests to compare values between patients. For serial samples from the same patient, one-way ANOVA was used to assess differences across cycles. Significance is indicated as: ns = not significant, *p* < 0.05 (*), *p* < 0.01 (**), *p* < 0.001 (***), *p* < 0.0001 (****).

However, a notable six-fold increase in regulatory T-cells (Tregs), expressed as a fraction of total CD4+ T-cells, was observed in the non-responder’s tumors (34.7%) relative to the responder (5.8%) (Fig. 4A, B). Over the course of treatment, Treg counts in the non-responder rose at cycle 2. In contrast, the responder exhibited a temporary increase in Tregs, peaking at the third cycle, followed by a decline by the final cycle. This pattern suggests an early immunosuppressive environment in the non-responder that may interfere with treatment efficacy, while the responder appears to maintain more effective immune surveillance.

Macrophages also differed significantly between the two patients’ tumors, constituting nearly a third (31.6%) of the non-cancer cells in the non-responder compared to 11.5% in the responder (Fig. 4A, C). Although macrophage percentages remained generally stable over time, the responder showed an occasional outlier spike at the first PIPAC cycle. Overall, these findings underscore the importance of immunosuppressive cell types, particularly Tregs and macrophages, in shaping differential therapeutic outcomes under PIPAC treatment.

## Discussion

To our knowledge, this is the first study to longitudinally follow single-cell molecular changes in LGSOC across multiple chemotherapy treatment cycles, providing unique temporal insights into treatment responses and failures. This exploratory single-cell analysis underscores how serial, repeated therapies influence tumor cell phenotype, pathway dynamics, and the immune microenvironment, thereby elucidating mechanisms of therapeutic response and resistance. The difference between responder and non-responder clearly illustrates that while effective inhibition of canonical growth and proliferation pathways can temporarily control tumor growth, compensatory mechanisms involving alternative signaling pathways, translational upregulation, metabolic adaptations, and immunosuppression may drive eventual therapeutic resistance. More broadly, multi-omics evaluation of LGSOC has been of increasing interest, as seen in a study by Gershenson et al. analyzing whole-exome sequencing, whole-transcriptome sequencing, and proteomics across 22 patients, which identified novel predictive biomarkers^13^. Our single-cell approach, while distinct in scope, complements such bulk multi-omics studies by providing temporal and cell-type–resolved insights into therapy-induced plasticity.

LGSOC is distinct among ovarian cancer histologic subtypes, characterized by inherent chemoresistance and MAPK and PIK3CA signaling pathways, making this study particularly relevant for uncovering specific chemoresistance mechanisms and elucidating the plasticity of these signaling pathways. Recent whole-exome sequencing studies have identified molecular subcohorts of LGSOC, notably showing that MAPK-mutant cases (KRAS, BRAF, NRAS) are more frequently associated with micropapillary stromal invasion and poorer survival^12^. In line with this, the non-responder harbored an NRAS mutation, consistent with the poor-prognosis MAPK-mutant subtype, whereas the responder had no significant targetable mutations. One of the core findings of this analysis is the differential response of Hallmark cancer pathways, including MYC, E2F targets, and the G2/M checkpoint, to chemotherapy treatments. In the non-responder tumors, these proliferation-associated pathways markedly increased alongside activation of KRAS signaling and the UPR, known to enhance tumor survival and chemoresistance under stressful conditions^14,15^. Notably, key stress-response mediators, including HSP90B1 and ATF4, both previously linked to chemotherapy resistance, were prominently upregulated^16–19^. Furthermore, strong induction of EMT and angiogenesis pathways suggests enhanced invasive capabilities and tumor adaptation to hypoxic or therapeutic stress, characteristics consistent with aggressive LGSOC progression^20–22^.

In contrast, the responder exhibited successful suppression of proliferation-associated pathways, especially MYC and E2F targets, indicating a reduction in translational activity and cellular growth capacity. However, alongside this suppression, we observed compensatory increases in alternative survival and growth pathways, notably PI3K/AKT, WNT/β-catenin, and Notch signaling. This dynamic pathway reprogramming aligns with earlier studies highlighting the role of these oncogenic pathways in survival and chemoresistance, where cancer cells rely on PI3K signaling to maintain survival under therapeutic stress^23–26^.

Further resolution of tumor heterogeneity via single-cell analyses revealed distinct subclonal archetypes dynamically evolving during treatment. The combined analysis across both patients’ tumors delineated two primary archetypes, characterized predominantly by either PI3K signaling and immune activity or metabolic pathways. Intriguingly, the responder’s tumor displayed an initial increase in the metabolic archetype, possibly reflecting an early adaptive attempt to evade growth suppression, followed by a reduction in this population with a resurgence of PI3K/immune-enriched cells by later cycles. This suggests a vulnerable state wherein metabolic adaptations could not sustain tumor growth under continued therapeutic pressure, leading to a positive clinical response, potentially due to the increased immune pathway signaling. By contrast, the non-responder maintained a stable archetype distribution, strongly enriched for PI3K signaling, alongside expansion of cells with a distinct translation and ribosome-associated signature, an archetype previously linked to aggressive and drug-resistant phenotypes in ovarian and other cancer^27,28^.

Our findings highlight the importance of the tumor immune microenvironment (TME) in shaping responses to chemotherapy. Notably, the non-responder showed significantly higher infiltration by Tregs and macrophages, both known to promote immunosuppression and support tumor survival^29^. Persistent high densities of these immune populations, especially Tregs, correlate with poor prognosis and therapeutic resistance^30^. In contrast, the responder patient exhibited low levels of Tregs, ultimately achieving a less immunosuppressive and potentially more immune-responsive environment. These observations strongly support the notion that therapeutic efficacy in LGSOC may be significantly enhanced by combination strategies that also target the immunosuppressive microenvironment, potentially through macrophage targeting or Treg depletion approaches^29,31^.

Collectively, this study underscores the complexity of LGSOC under therapeutic pressure, demonstrating that single-pathway or single-agent strategies are likely insufficient to achieve durable disease control. While MEK inhibitors (e.g., Trametinib) remain the standard of care, many patients ultimately progress^32^. Multi-omics analyses of MEKi-resistant models reveal alterations in both MAPK and PI3K-AKT-mTOR pathways^33^. These observations suggest that PI3K activation may provide a compensatory escape route under MAPK blockade. While PI3K activation in MEKi-resistant models suggests a potential resistance mechanism, it remains uncertain whether these alterations are true drivers or adaptive changes. Early clinical trials of PI3K inhibitors (e.g., GDC0941, XL147, BKM120) have shown modest signals of activity in ovarian cancer, but overall responses have been limited, particularly in unselected populations^34^. These findings highlight the need for biomarker-guided approaches.

In addition to PI3K/MAPK crosstalk, the Wnt/β-catenin pathway promotes cancer stem cell self-renewal and chemoresistance across ovarian cancer subtypes^35^. In HGSOC, β-catenin inhibition sensitizes tumors to cisplatin and reduces tumor sphere formation, demonstrating a causal role in maintaining chemoresistance^36^. Although less well defined in LGSOC, these findings suggest that Wnt-driven stemness programs may similarly contribute to therapeutic persistence.

Together, these insights emphasize the need to distinguish causal drivers of resistance from adaptive changes, and to leverage real-time molecular profiling to guide rational combinations, such as RAF/MEK plus PI3K/AKT inhibitors (e.g. in clinical trials, GOG-3097, RAMP-201), as well as emerging strategies like CDK4/6 inhibitors with hormonal agents^37–39^. Dynamic, adaptive treatment approaches will be critical to intercept subclonal evolution and acquired resistance.

Despite these compelling insights, our findings are inherently exploratory, limited by a small cohort size, partial immune profiling, and few longitudinal sampling points, particularly in the non-responder patient due to disease progression and withdrawal from treatment. The study’s conclusions are constrained by being drawn from only two patients, which limits statistical power and generalizability, and potential confounding from demographic and clinical covariates (e.g., prior therapies, mutational status) cannot be excluded. Accordingly, the findings should be framed as hypothesis-generating. More extensive and spatially resolved sampling, coupled with spatial-omics technologies, will be critical for capturing the full breadth of tumor and immune evolution in larger, comprehensive studies.

In conclusion, by revealing detailed cellular and molecular reprogramming mechanisms in recurrent LGSOC under PIPAC treatment, this study provides foundational insights into tumor resilience and therapeutic escape. It highlights the necessity of multi-targeted, adaptive therapeutic approaches to effectively manage the inevitable evolutionary responses of tumor cells in this challenging cancer type.

## Methods

### Study design and ethical considerations

This is a non-escalating, feasibility, single-arm Phase I clinical trial designed to assess the safety of PIPAC delivered at intraperitoneal doses of 10.5 mg/m² for cisplatin and 2.1 mg/m² for doxorubicin. Conducted in compliance with the ethical standards set forth in the Belmont Report and the Declaration of Helsinki, the trial has secured approvals from the Institutional Review Board at City of Hope (IRB #19184). This trial was registered at ClinicalTrials.gov (registration number: NCT04329494, date of registration 2020-08-21). All patients completed written documentation of informed consent, including the use of data and images for publication. Patients: Adult patients ≥18 years old with histologically confirmed ovarian carcinoma with peritoneal carcinomatosis based on either cross-sectional imaging or diagnostic laparoscopy who had progressed on at least one evidence-based chemotherapeutic regimen were included if their Eastern Cooperative Oncology Group (ECOG) performance status was ≤2, there were no contraindications to laparoscopic surgery or aerosol therapy, intraoperative laparoscopic findings showed PIPAC access was feasible, there was no evidence of impending bowel obstruction and ≤5 L of ascites, and patient was not a candidate for cytoreduction and HIPEC. Exclusion criteria included receiving prior maximum cumulative doses of anthracyclines and/or anthracenediones. See Supplementary Document S2 for complete eligibility and exclusion criteria.

Study Design: This is a nonrandomized, uncontrolled, single-arm, Phase I clinical trial without dose escalation to establish the safety of PIPAC-cisplatin/doxorubicin (PIPAC-CD) in the U.S. A total of 15 ovarian cancer patients were enrolled in this study and underwent PIPAC-CD, with clinical outcome results described previously (Nakamura et al, Annals of Surgical Oncology, 2025). From this trial, the best responder (Pt #2) and a non-responder (Pt#1) with available tissue biopsies were selected for single-cell omics.

Participants were scheduled for PIPAC administration every six weeks, with three planned treatments, unless they experience severe adverse events (AEs), dose-limiting toxicities (DLTs), disease progression, or choose to withdraw. Patients who experienced clinical benefit from PIPAC (e.g., partial response) were offered up to an additional 3 cycles of PIPAC (total of 6 cycles). The primary objective was to establish the safety profile of the specified intraperitoneal doses of cisplatin and doxorubicin by closely monitoring AEs, DLTs, disease progression, and patient withdrawals.

### PIPAC procedure

During each cycle of PIPAC, the laparoscopic open entry technique was performed, and two additional laparoscopic ports were placed under direct visualization. Visual assessment of tumor burden was recorded using the Peritoneal Carcinomatosis index (PCI), and biopsies from all accessible quadrants were collected for the Peritoneal Regression Grading Score (PRGS) (18). Biopsy sites were selected by the surgeon based on the largest and most suspicious-appearing tumor lesions. After biopsies were collected, PIPAC was performed using standardized port placement, with aerosolized intraperitoneal cisplatin 10.5 mg/m^2^ in 150 mL NaCl 0.9% and doxorubicin 2.1 mg/m^2^ in 50 mL NaCl 0.9% delivered using a high-pressure injection (Medrad Stellant injector, Bayer Corporation) and a Nebulizer (REGER Medizintechnik GmbH, Villingendorf, Germany) at an average of 200 psi at 0.5 mL/s with a maximum of 300 psi. This was followed by a 30-min pneumoperitoneum at 12 mmHg containing the aerosolized chemotherapy within the abdomen at room temperature prior to release of the pneumoperitoneum and evacuation of the aerosolized chemotherapy. No additional surgical interventions or tumor resections were performed during the PIPAC procedure. PIPAC was repeated at 4–6-week intervals for a planned total of three treatments unless severe adverse events (AE), dose-limiting toxicity (DLT), disease progression, or patient withdrawal from the study occurred.

Endpoints: The primary endpoint was to evaluate the safety of PIPAC-CD by assessing DLTs and the incidence of treatment-related AEs. AEs were assessed at intervals of every 4–6 weeks for up to 18 weeks using the Common Terminology Criteria for Adverse Events version 5.0 (CTCAE v5.0). Following treatment completion (≥2 PIPACs), patients were evaluated every 12 weeks. Secondary endpoints evaluated the efficacy of PIPAC utilizing changes in computed tomography (CT) imaging, RECIST version 1.1, pathologic PRGS of intraoperative biopsies taken each cycle, and intraoperative PCI. RECIST by CT imaging was evaluated after cycle 2 and at the completion of treatment. PCI was calculated by the same surgeon for each patient across all PIPAC cycles received. PRGS was reported as the average of all quadrant biopsies obtained during each cycle of PIPAC. CA-125 tumor markers were evaluated in patients at the beginning of each cycle.

### Tissue collection

Tumor peritoneum and non-tumor involved (normal) samples were collected based on tumor-affected spatial quadrants of the abdomen in which they were collected (right/left, upper/lower) before and after each PIPAC treatment. The first tumor collection in each patient at their first PIPAC cycle was a baseline tissue biopsy prior to any intraperitoneal chemotherapy exposure, and considered a “time point 0” evaluation. A total of two and six cycles of PIPAC treatment were performed for the non-responder and the responder patient, respectively. While three cycles were planned under protocol guidelines, the non-responder patient progressed after two cycles and discontinued the treatment protocol; the responder patient had a partial response and thus clinical benefit, and therefore continued to three additional cycles after 3 cycles of protocol therapy for a total of 6 cycles. Tissue samples were cut into pieces of 2–5 mm^2^ in size and frozen at −80 °C in 50% RPMI-1640 (Gibco, Cat # 11875) + 40% heat-inactivated fetal bovine serum (hiFBS, Millipore Sigma, Cat # 12306C) + 10% DMSO (Fisher Scientific, Cat. # D2650).

### H&E

Tissue specimens underwent standard formalin fixation followed by routine paraffin-embedded processing. Histological sections were prepared and stained using standard hematoxylin and eosin (H&E) staining procedures to assess tissue morphology.

### Nuclei Isolation

Processing on ice, cryopreserved tissue was thawed in a 37 °C water bath and quickly rinsed in 5 mL a modified cytoprotective buffer containing components from Vrselja et al BEx Perfusate Buffer^40^ containing 1× PBS (Gibco, Cat# 10010) supplemented with 539 nM Necrostatin-1, 996 nM HPN-07, 317 nM Sodium 3-Hydroxybutyric Acid, and 77.9 nM Q-VD-OPh hydrate (Sigma Aldrich, Cat# N9037, SML2163, 54965, and SML0063 respectively). Nuclei were isolated as developed by Gao et al.^41^ and modified as previously described^42,43^. Briefly, tissue was minced and incubated for 15 min in a (4:1) Igepal CA-630 Lysis Buffer to DAPI buffer supplemented with fresh 0.2 U/µL SUPERase⋅In RNase Inhibitor (Invitrogen, Cat. # AM2694) on ice at 4 °C to release nuclei. Lysate was filtered through a 40 μm mesh cell strainer (Greiner Bio-one, Cat# 542040) and nuclei were washed 1× with 2 mL of (4:1) Igapel CA-630: DAPI Lysis Buffer and 2× times by washing and final resuspension in 1× PBS + 1.0% RNase free BSA (Millipore Sigma, Cat # 26615-25 mL) + 0.2U/µl SUPERase-In RNase inhibitor (Invitrogen, Cat # AM2696) centrifugation at 500 × *g* for 5 min at 4 °C between washes. The final nuclei suspension was filtered through a 40 μm mesh cell strainer. Nuclei were counted on a hemocytometer by DAPI fluorescence using an Invitrogen Countess II equipped with a DAPI filter cube and maintained at 4 °C prior to snRNA-Seq.

### Single-nuclei RNA sequencing

Gel Bead-In emulsions (GEM) Single-nuclei suspensions were prepared using the 10X Genomics Chromium Single Cell System with Chromium Single Cell 3′ V3 kit (Cat# PN-1000075) for the non-responder and Chromium Next GEM Single Cell 3′ V3.1 Dual Index kit (Cat # PN-1000268) for the responder. Nuclei were diluted to a concentration of 500–2000 nuclei/µl using a target recovery of 5000 cells per sample. The barcoding, reverse transcription, and library preparation steps were carried out following the manufacturer’s protocol. The resulting cDNA libraries were sequenced using Illumina NovaSeq 6000 instruments with 150-bp paired-end reads at a median depth of 34,000 reads per cell.

SnRNA-seq was chosen over scRNA-seq because, in solid tissue, nuclei-based approaches achieve higher recovery rates and minimize dissociation-induced cell stress and loss, thereby preserving malignant and stromal populations, albeit with reduced representation of immune cells^44–48^.

### Data preprocessing and quality control

The raw 10× reads were preprocessed using CellRanger, and the reads were aligned to the GRChg38 reference genome using Bioinformatics the ExperT SYstem and CellRanger v.3.0.2 pipelines^49^. Read counts were extracted using featureCounts^50^. The raw count matrix was then processed to remove doublets and filtered using the Seurat pipeline using the following criteria: nFeature_RNA > 300, nFeature_RNA < 8000, and percent.mt <5, assuming a 4% doublets rate, resulting in 18,351 high-quality cells^51^.

### Cell type annotation and dimensionality reduction

Cell types were annotated using reference-based cell-type annotations from SingleR^52^. The annotations were then validated by using projecting the harmony-integrated dataset on Uniform Manifold Approximation and Projection (UMAP) and utilizing canonical gene markers for various cell types including immune cells (PTPRC), epithelial cells (EPCAM, ESR1, KRT7, KRT8, KRT18, KRT19), macrophages/monocytes (ITGAM, CD163, TYROBP, CSF1R), T-cells (CD247, BCL11B, GRAP2, THEMIS, TNFRSF18), B-cells (MS4A1, CD19), fibroblasts (THY1, CDH11, COL1A1, COL1A2, COL5A1, FBLN1, FBLN2, PDGFRA, PDPN), endothelial cells (CD34, ESAM, VWF), adipocytes (CIDEA, DGAT2, PLIN4).

### Identification of malignant cells

To distinguish between malignant and normal cells, CopyKat was used to obtain copy number alteration (CNA) profiles of putative malignant cells, which were previously annotated as epithelial cells by SingleR and single-gene markers^53^. Cell types other than epithelial and fibroblasts were used as a reference using the following parameters: ngene.chr=5, win.size=50, KS.cut=0.1, distance = “euclidean” (Supplementary Fig. S8A, B). Cells that could not be confidently classified by CopyKat and were labeled as not.defined were further analyzed using a supervised classification approach. Specifically, a SingleR model was trained on cells confidently labeled as diploid or aneuploid by CopyKat, and then applied to reclassify the not.defined population. Model performance was evaluated using multiclass AUC (area under the ROC curve), and predictions were accepted only if the model achieved an AUC > 0.7. This approach allowed for recovery and refinement of CNA-based annotations in low-confidence cells, enabling a more complete classification of the malignant versus normal cell population.

### Tumor microenvironment assessment by multiplex immunofluorescence

Formalin-fixed paraffin-embedded (FFPE) tissue specimens were cut at 5 µm sections and baked onto glass slides. The FFPE slides were then deparaffinized in xylene and rehydrated in decreasing ethanol concentration washes. Heat-induced antigen retrieval was performed using boiling AR9 buffer, 10× (pH 9) (Akoya Biosciences) in a microwave oven for 20 min. Blocking was performed for 10 min using Antibody Diluent with Background-Reducing Components (Agilent). Primary antibodies were incubated for 1 h on a shaker at room temperature, followed by a 10-min incubation of horseradish peroxidase (HRP)-conjugated secondary antibody (Mouse HRP-Polymer, Biocare Medical). Immunofluorescent labeling of antibodies was achieved using the Opal 7-color fluorescence IHC Kit (Akoya Biosciences) at a 1:100 dilution for 10 min. Slides were serially stained with the microwave incubation, acting to remove previous antibodies while simultaneously exposing the next epitope of interest. After staining the final marker, cell nuclei were stained with DAPI (Akoya Biosciences), and the slides were mounted with ProLong Gold Antifade Reagent (Thermo Fisher Scientific). Primary antibodies were as follows: CD3 (clone LN10, Leica), CD8 (clone 4B11, Leica), CD68 (clone KP1, Biocare), FOXP3 (clone 236A/E7, Abcam), and pan-cytokeratin (clone AE1/AE3, Dako). Tissue slides were scanned using the Vectra 3.0 automated quantitative pathology imaging system (Akoya Biosciences). Images were spectrally unmixed with inForm® tissue analysis software (Akoya Biosciences), and component TIFFs were exported for whole slide analysis using QuPath software^54^.

### Phenotype assignment and pathway analysis

We first normalized the single-nuclei RNA-seq data for read depth, gene length, and GC-content using ZINB-WaVE^55^. Afterward, to assign pathway-level phenotypes to individual cells, we computed single-sample gene set enrichment scores using the GSVA package^56,57^. Specifically, we leveraged both the Hallmark and C2 gene set collections (*n* = 5513), yielding pathway enrichment scores (ssGSEA) at the single-nuclei level.

### Pathway and gene enrichment analysis over time

We next modeled these pathway ssGSEA scores, and in parallel, selected gene expression levels across sequential timepoints to capture dynamic trends. In particular, each pathway or gene was treated as a response variable in a linear model, with time point included as the explanatory variable. The resulting estimates and *p*-values were corrected for multiple hypothesis testing via the Benjamini–Hochberg procedure. Any pathway or gene surpassing the chosen false discovery rate (FDR) threshold (0.05) was considered significantly enriched or differentially expressed over time.

### Archetype analysis

To identify cancer cell archetypes, we first combined patient-derived cells and performed SVD on their ssGSEA scores, retaining only those components explaining 90% of the variance. Before proceeding, batch effects were corrected by scaling the SVD components to unit variance and calculating the absolute distances between group means for each component; the SVD component with the largest distance between groups, indicative of significant batch effects, was removed from further analyses. The same analysis was performed independently for each patient, but without correcting for batch effects. Using this adjusted reduced-dimensional representation, we determined the optimal number of archetypes (clusters) by performing consensus clustering with k-means clustering (Euclidean distance), evaluating solutions for cluster numbers (k) ranging from 2 to 10. Stability of each clustering solution was assessed by repeatedly subsampling 80% of the cells for 100 iterations, calculating consensus scores based on how frequently pairs of samples co-clustered across iterations. The proportion of ambiguous clustering (PAC) metric was then computed by identifying the fraction of these consensus scores falling within an ambiguous intermediate range (0.1–0.9); the optimal number of clusters (k = 2) was selected based on maximum stability, defined as (1 - PAC). The selected number of clusters (k) was subsequently used to cluster cells using the Louvain algorithm, implemented via Seurat’s FindNeighbors and FindClusters functions, which identify clusters based on shared nearest neighbor modularity optimization.

To characterize the biological functions of the archetypes, we conducted Differential Pathway Expression (DPE) analysis. The DPE analysis employed the Wilcoxon test to identify significantly enriched pathways within each archetype, selecting the top 20 genes per cluster based on statistical significance and differential expression levels.

### Statistical analysis

Statistical analyses were performed using two-sided tests. Multiple comparisons were corrected using the Benjamini–Hochberg procedure to control the FDR, with an adjusted *p*-value threshold of 0.05 considered significant^58^. All statistical analyses were conducted with R, and FDR adjustments were applied to account for multiple hypothesis testing.

## Supplementary information

41698_2025_1182_MOESM1_ESM.

## Data Availability

The Seurat objects, including count matrices and metadata supporting the findings of this study, are openly available on Figshare (10.6084/m9.figshare.28836257).

## Code availability

The analytical code used in this study can be accessed via GitHub at https://github.com/ibishara/PIPAC.
