# Supplementary material for: Divergent oncogenic signaling and immune microenvironment changes in low-grade serous ovarian cancer patients undergoing intraperitoneal chemotherapy
Source: NPJ Precis Oncol. 2025 Dec 14;10:1. doi: 10.1038/s41698-025-01182-3 (PMC12764849; doi:10.1038/s41698-025-01182-3)
Supplement: Supplementary file 1 — 41698_2025_1182_MOESM1_ESM. [file 41698_2025_1182_MOESM1_ESM.pdf]

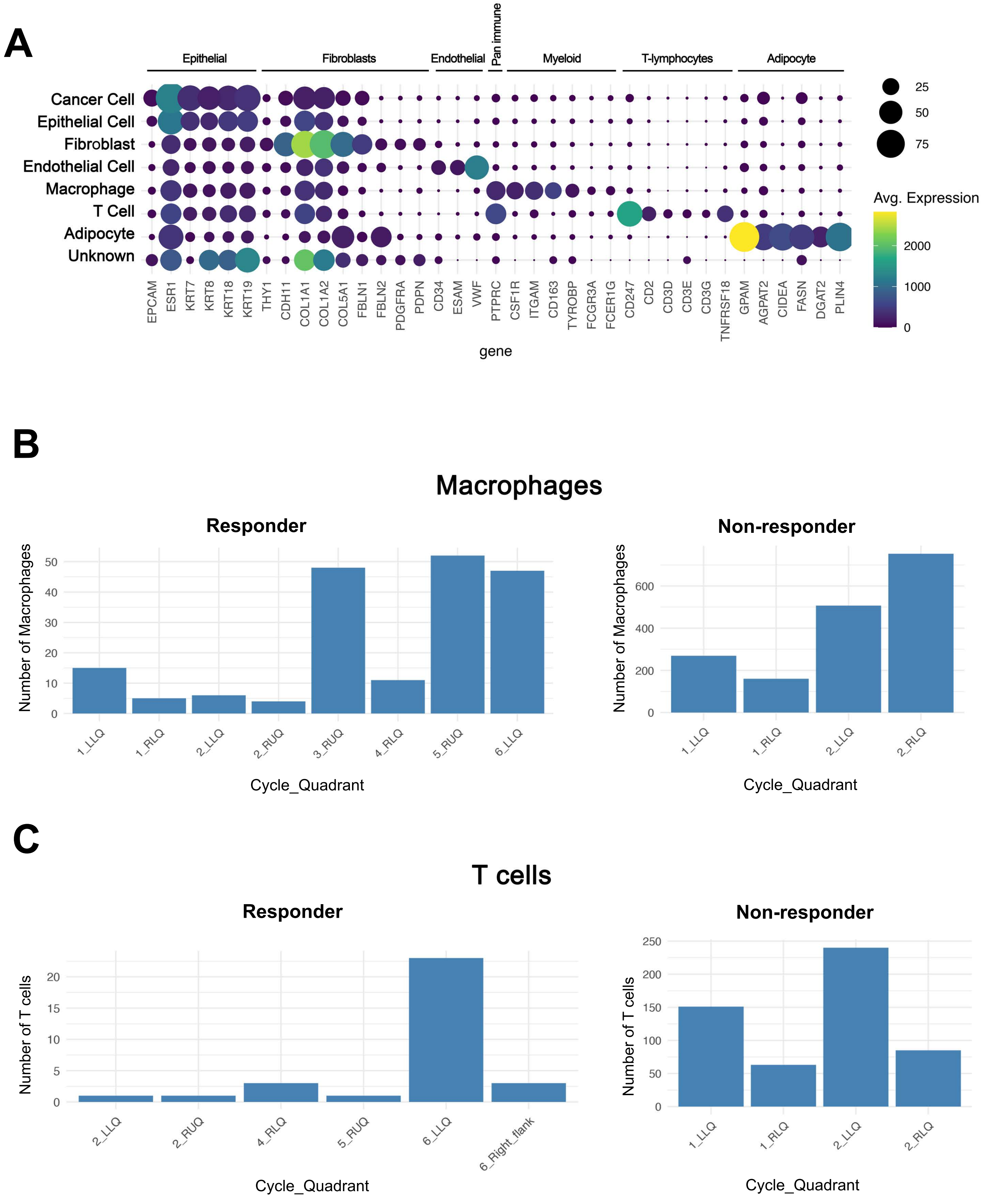

Supplemental Figure S1. Cell type annotation and immune cell quantification.

(A) Dot plot showing reference-based cell type annotations obtained using SingleR. The expression of canonical gene markers across major cell types is displayed. The size of each dot represents the proportion of cells expressing the gene, while color indicates average CPM-normalized expression level. (B) Bar plots depicting the number of macrophages across distinct treatment cycles and anatomical quadrants (LUQ: left upper quadrant; LLQ: left lower quadrant; RLQ: right lower quadrant; RUQ: right upper quadrant) for responder (left) and non-responder (right) patients. Non-responders demonstrate consistently elevated macrophage counts across samples, whereas responders exhibit markedly reduced macrophage presence. (C) Bar plots quantifying the number of T-cells at different timepoints and quadrants in responder (left) and non-responder (right) patients. The non-responder displays a greater presence of T-cells, while the responder has very few detected T-cells across all timepoints and regions.

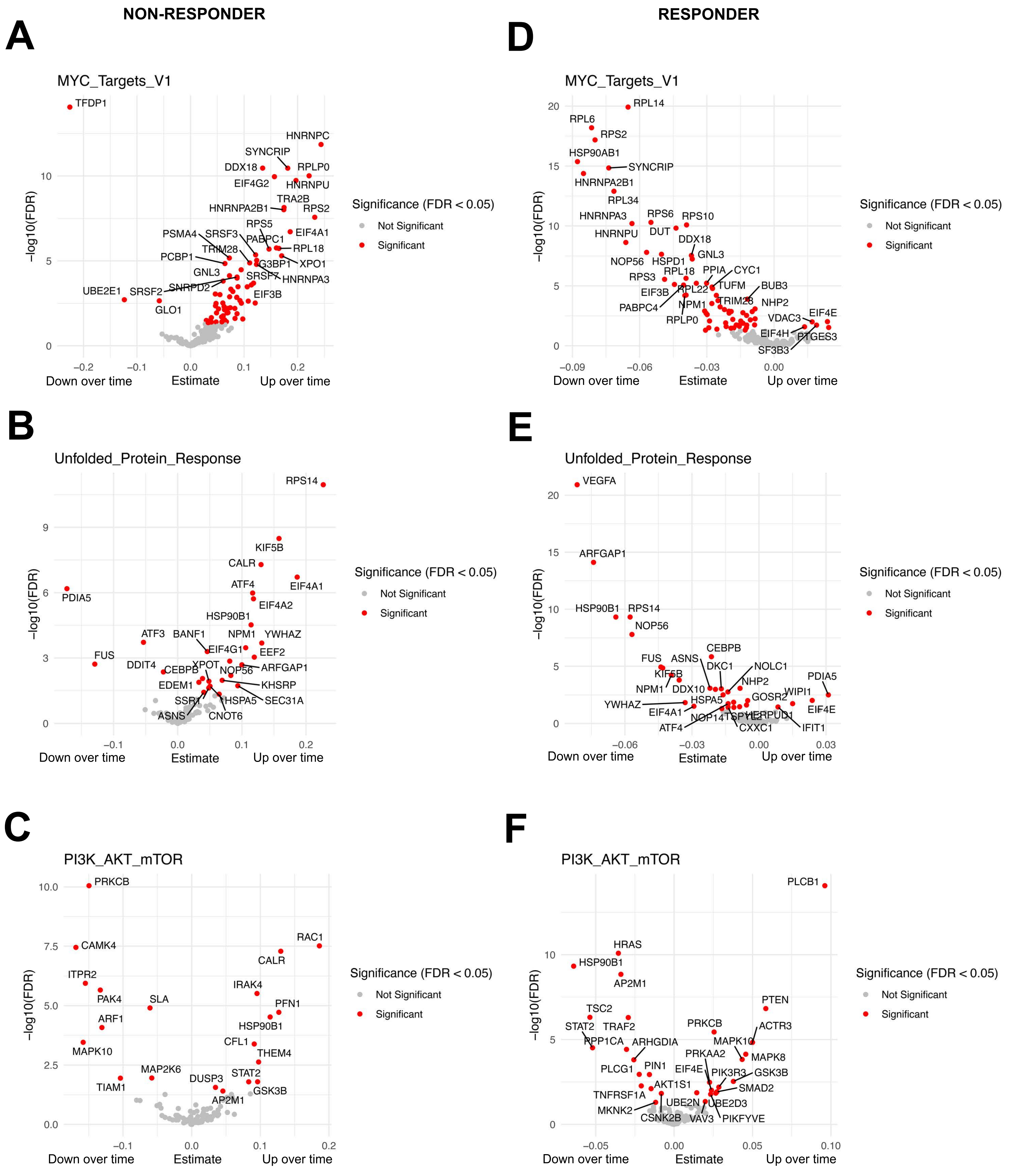

Supplemental Figure S2. Gene-level alterations within key pathways during PIPAC treatment.

(A-C) Volcano plots showing temporal changes in gene expression within key pathways in the non-responder patient. (A) MYC target pathway, highlighting significant upregulation of genes involved in protein synthesis (e.g., RPL and RPS gene families) and stress adaptation (HSP90B1, ATF4), suggesting enhanced tumor growth and resistance mechanisms. (B) UPR, showing increased expression of genes linked to stress adaptation. (C) PI3K-AKT-mTOR pathway, highlighting upregulation of key signaling mediators (e.g., PRKCB, RAC1, MAPK10), reflecting sustained activation of oncogenic signaling. (D-F) Volcano plots of the same pathways in the responder patient. (D) MYC target genes involved in protein synthesis are significantly downregulated. (E) UPR-related stress genes (HSP90B1, ATF4) show decreased expression, consistent with diminished stress adaptation and growth reliance. (F) PI3K-AKT-mTOR pathway, revealing a shift in pathway activity with decreased HRAS expression and increased PTEN, while PIK3R3 and MAPK10 are upregulated, suggesting modulation of survival signaling under treatment pressure. FDR < 0.05 was considered significant.

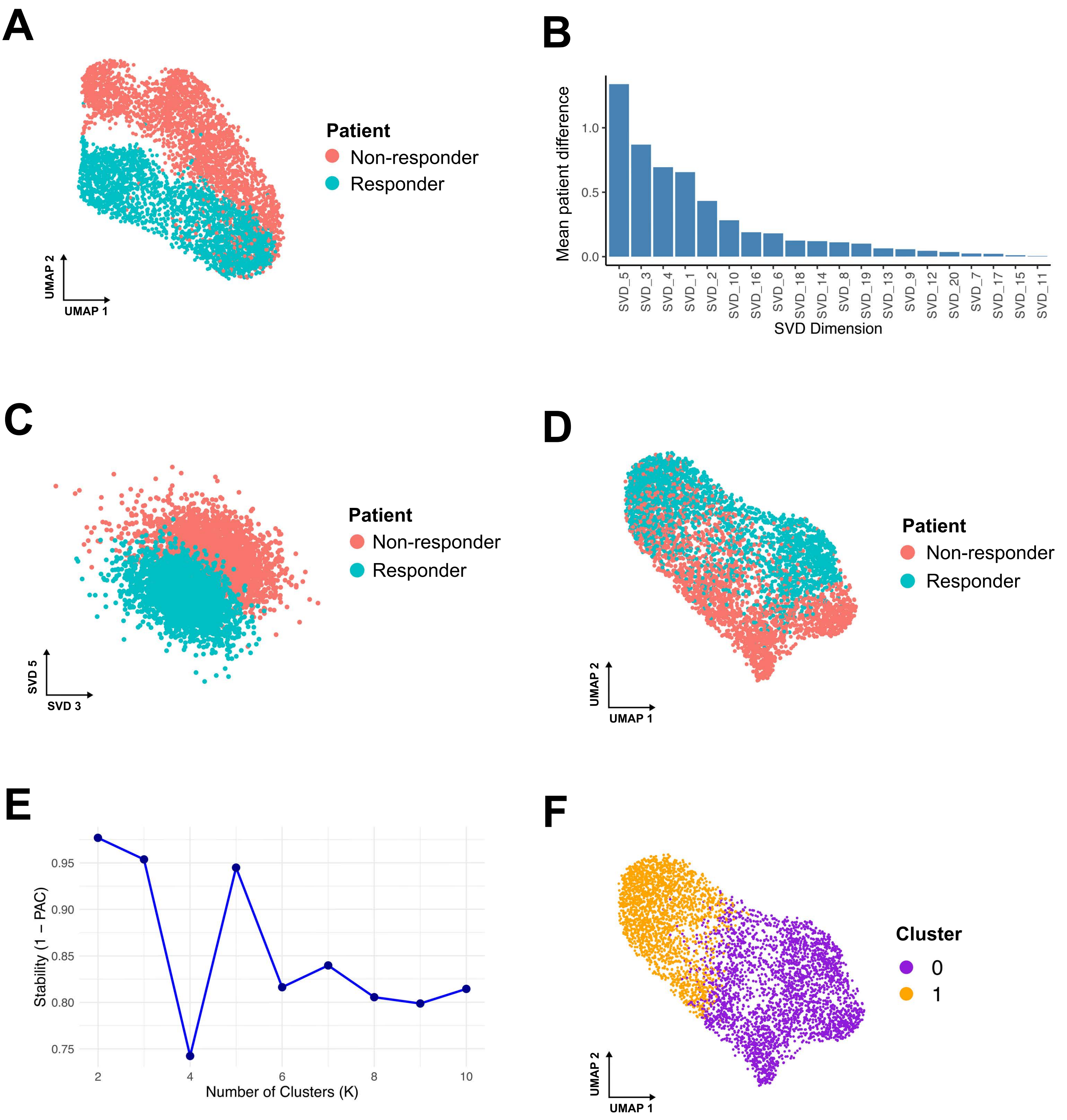

Supplemental Figure S3. Batch effect assessment and archetype determination via SVD and clustering stability analysis.

(A) UMAP visualization of single-nuclei data derived from combined patients prior to batch effect correction, demonstrating clear patient-specific grouping. (B) Bar plot showing the mean absolute difference between patient groups for each of the top SVD dimensions, identifying dimensions contributing significantly to batch effects. (C) Scatter plot of two selected SVD dimensions (SVD3 and SVD5) illustrating patient-driven differences in dimensionality prior to correcting for batch effects. (D) UMAP visualization after removing the top SVD component associated with significant batch effects, showing improved integration of the data from both patients. (E) Consensus clustering stability analysis evaluating the stability of clustering solutions across a range of potential cluster numbers (K = 2–10). Stability, calculated as (1 - PAC), identifies K = 2 as the most stable solution. (F) UMAP visualization of the final clustering of cells into two archetypes using the Louvain algorithm following the determination of optimal cluster number (K = 2).

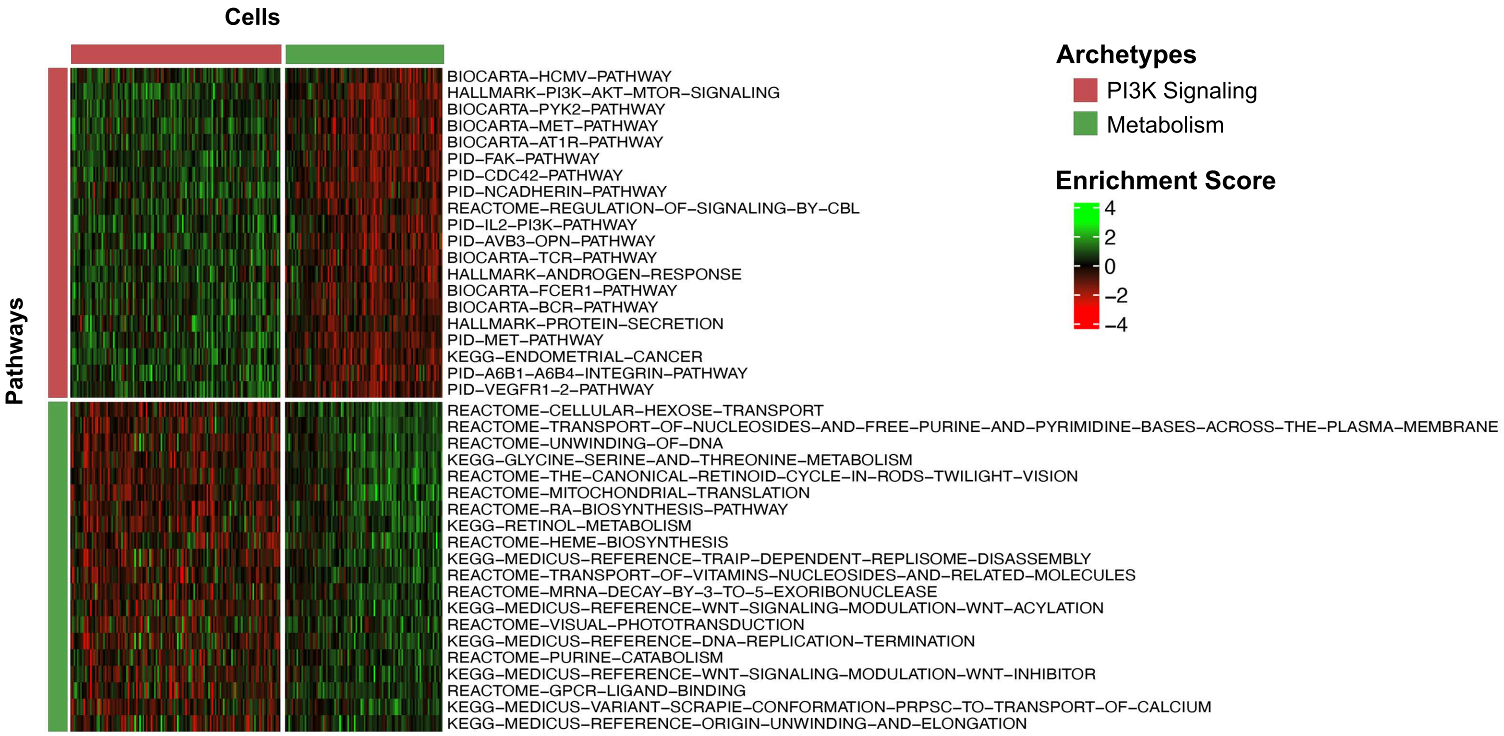

Supplemental Figure S4. Detailed pathway enrichment heatmap for the two archetypes identified in the combined patient analysis.

Heatmap displaying the enrichment scores of the top 20 pathways significantly enriched in each archetype, identified using Differential Pathway Expression (DPE) analysis and the Wilcoxon Rank Sum test. Cells are grouped by their respective archetype assignments: the PI3K signaling archetype prominently shows enrichment for PI3K-related signaling pathways (including PI3K-AKT-mTOR, IL-2-PI3K, T-cell receptor, B-cell receptor, and integrin signaling), immune response pathways, and other receptor-mediated signaling cascades. The metabolism archetype displays enrichment across diverse metabolic pathways, including amino acid metabolism, nucleotide metabolism, heme biosynthesis, retinoic acid metabolism, mitochondrial translation, nucleoside transport, and carbohydrate transport, among others. The heatmap illustrates clear pathway-based differentiation between the identified cellular archetypes.

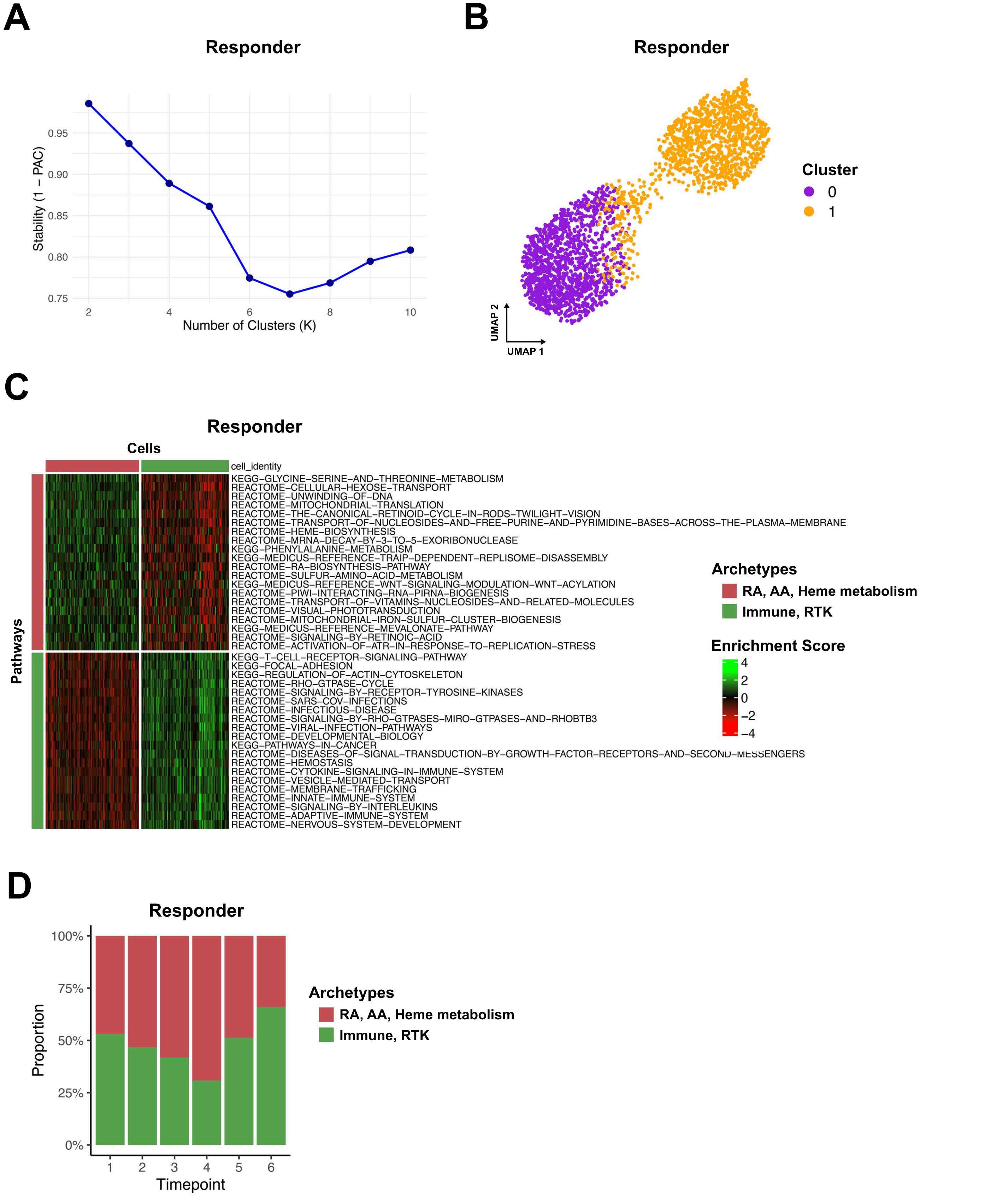

Supplemental Figure S5. Individual archetype analysis for the responder patient.

(A) Consensus clustering stability plot identifying the optimal number of archetypes (clusters) as K = 2 based on maximum stability (1 - PAC). (B) UMAP visualization of individual cells from the responder patient, colored by their assigned archetype clusters determined using the Louvain algorithm. (C) Heatmap showing the top 20 significantly enriched pathways for each archetype identified in the responder patient. The first archetype demonstrates enrichment of metabolic pathways, including retinoic acid (RA), amino acid (AA), heme metabolism, nucleoside transport, and mitochondrial translation. The second archetype highlights enrichment in receptor tyrosine kinase (RTK) signaling and immune-related pathways, including T-cell receptor signaling, cytokine signaling, and adaptive immune responses. (D) Bar plot illustrating the dynamic changes in cell proportions belonging to each archetype across six treatment cycles in the responder patient. The metabolism archetype initially increases from approximately 50% to 70% before sharply declining to around 30%, coinciding with a corresponding expansion of the RTK/immune archetype.

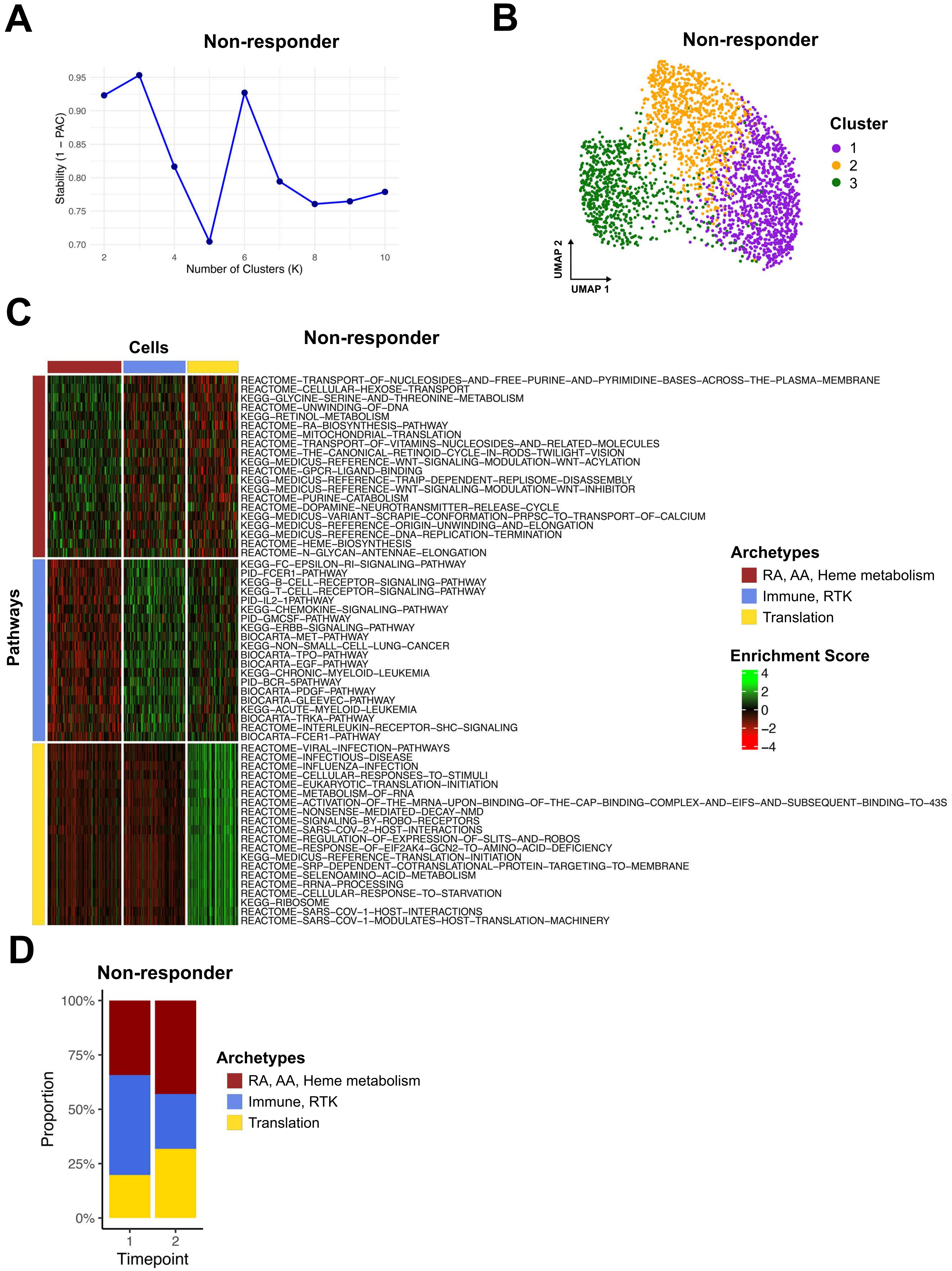

Supplemental Figure S6. Individual archetype analysis for the non-responder patient.

(A) Consensus clustering stability plot indicating the optimal number of archetypes (clusters) as K = 3 based on the highest clustering stability (1 - PAC). (B) UMAP visualization of individual cells from the non-responder patient, colored by their assigned archetype clusters determined using the Louvain algorithm. (C) Heatmap presenting the top 20 significantly enriched pathways for each archetype identified in the non-responder patient. The three archetypes include (1) metabolism archetype enriched for retinoic acid (RA), amino acid (AA), heme metabolism, and nucleoside transport pathways; (2) receptor tyrosine kinase (RTK)/immune archetype enriched for immune signaling pathways such as T-cell receptor, B-cell receptor signaling, cytokine and interleukin signaling; and (3) translation archetype characterized predominantly by protein translation, ribosomal biogenesis, and translational initiation pathways. (D) Stacked bar plot showing proportions of cells assigned to each archetype across two treatment cycles in the non-responder patient. Notably, the RTK/immune archetype decreases, while the translation archetype expands significantly, accompanied by a moderate increase in the metabolism archetype.

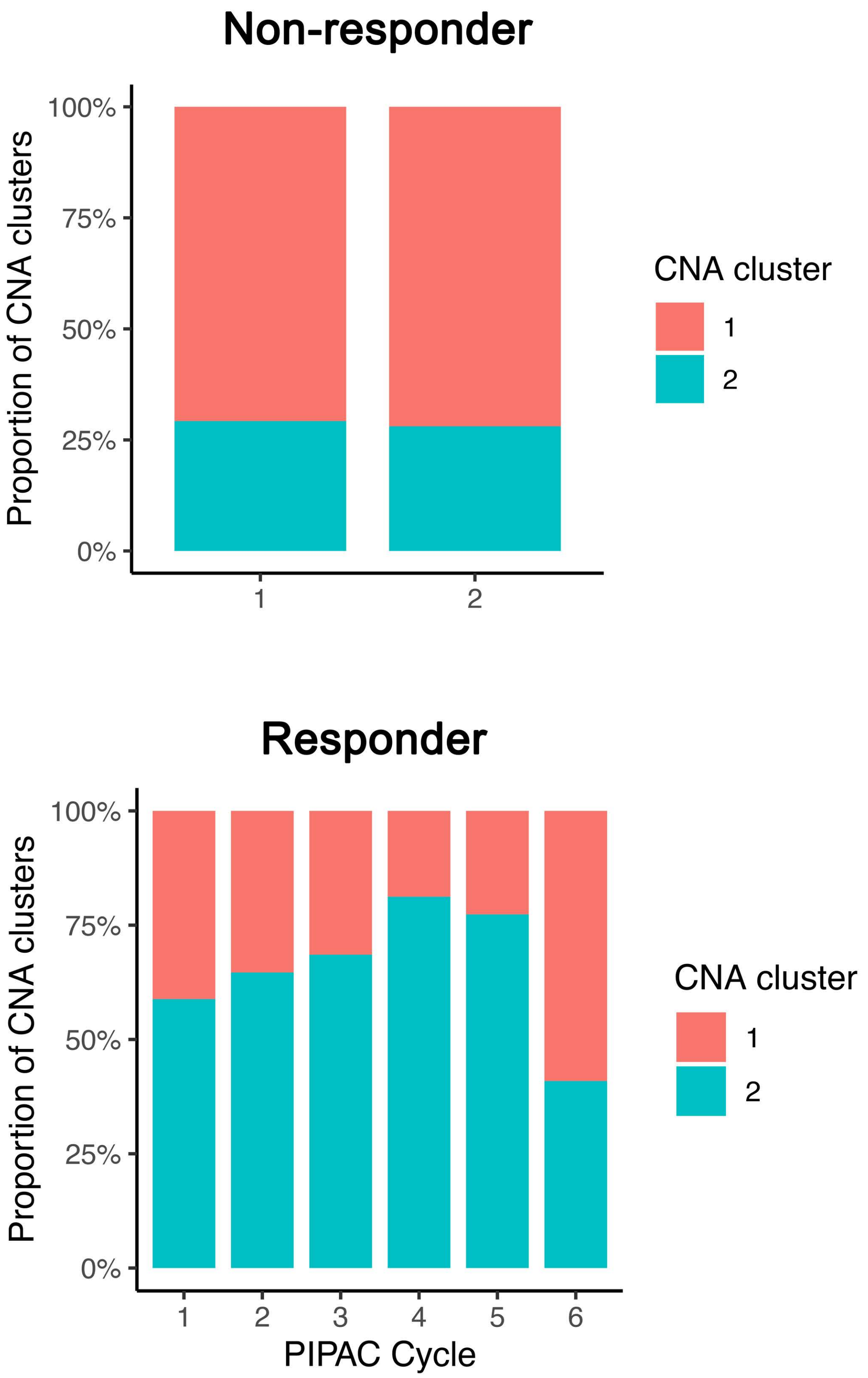

Supplemental Figure S7. Copy number alteration (CNA)-based subclonal dynamics of malignant cells across treatment cycles

Malignant cells were clustered into two subclones (CNA clusters 1 and 2) using K-means (K=2) on copyKat inferred CNA profiles. Stacked bar plots display the relative proportions of the two CNA clusters across treatment cycles for the non-responder (top) and the responder (bottom) patients.

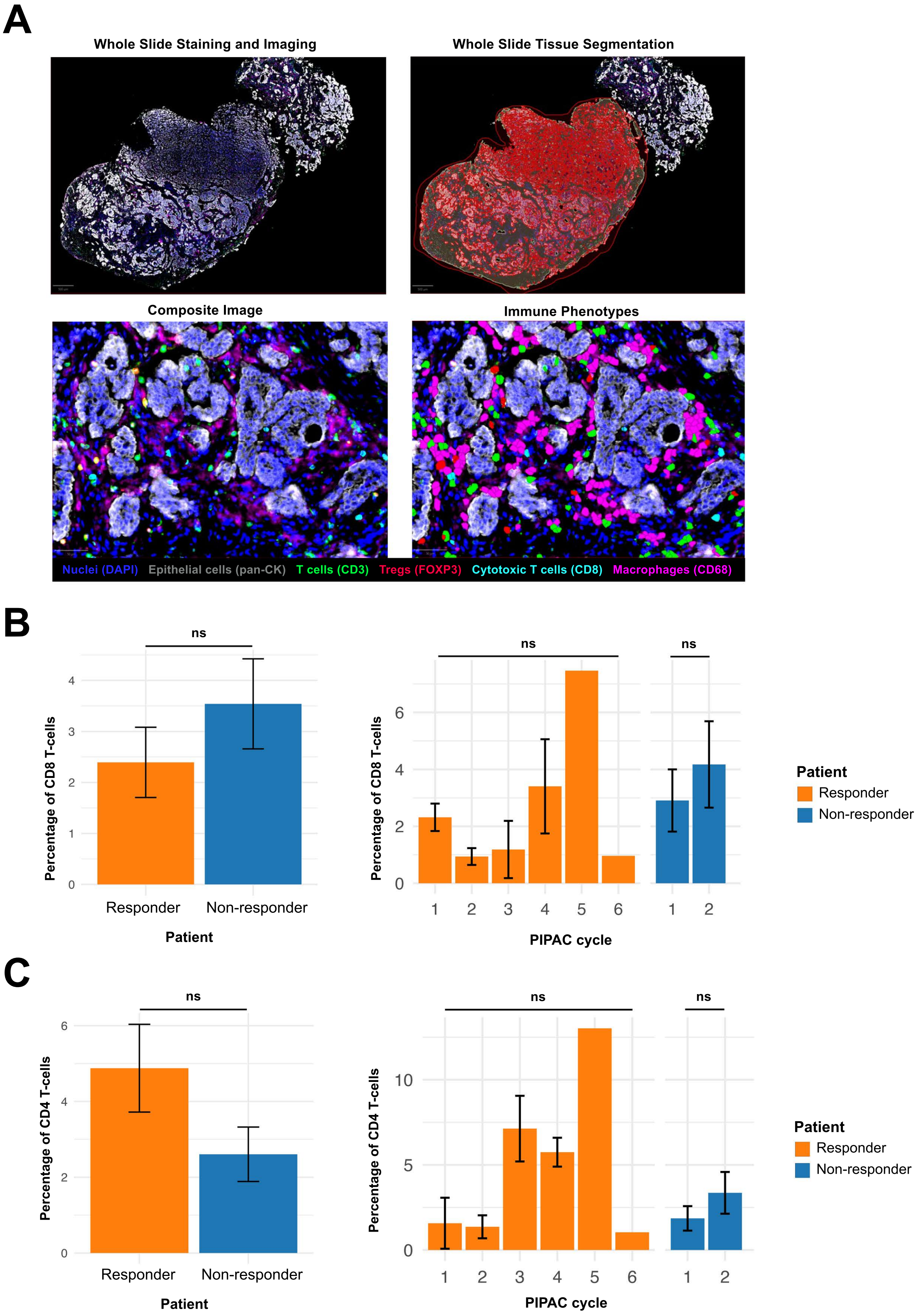

Supplemental Figure S8. Immunostaining analysis of T-cell populations in responder and non-responder patients.

(A) FFPE Tissue specimens were assessed via multiplex immunofluorescence. Whole slides were stained and imaged. All identified quality tumor tissue was selected for immune quantification. Tissue segmentation was performed to distinguish cancer islands (red) from tumor stroma (green). A machine learning approach was taken to classify cells as positive or negative for staining of immune markers. Resulting immune phenotypes were normalized as densities across patient tissues to the area of stroma tissue assessed. (B) Bar plots showing the percentage of CD8+ T-cells in responder and non-responder patients. Left panel represents the overall CD8 T-cell proportions averaged across all sampled timepoints. The right panel depicts temporal changes across individual PIPAC treatment cycles, highlighting increased CD8 T-cell proportions in the responder patient, particularly notable at cycle 5, compared to more consistent proportions in the non-responder. (C) Bar plots displaying overall and temporal proportions of CD4 T-cells. Similar to CD8 T-cells, the responder patient shows variable proportions across cycles, peaking at cycle 5, whereas the non-responder demonstrates relatively stable CD4 T-cell levels over the two treatment cycles. Statistical analysis was performed using unpaired two-tailed t-tests to compare values between patients. For serial samples from the same patient, one-way ANOVA was used to assess differences across cycles. Significance is indicated as: ns = not significant, $p < 0.05$ (*), $p < 0.01$ (**), $p < 0.001$ (***), $p < 0.0001$ (****).

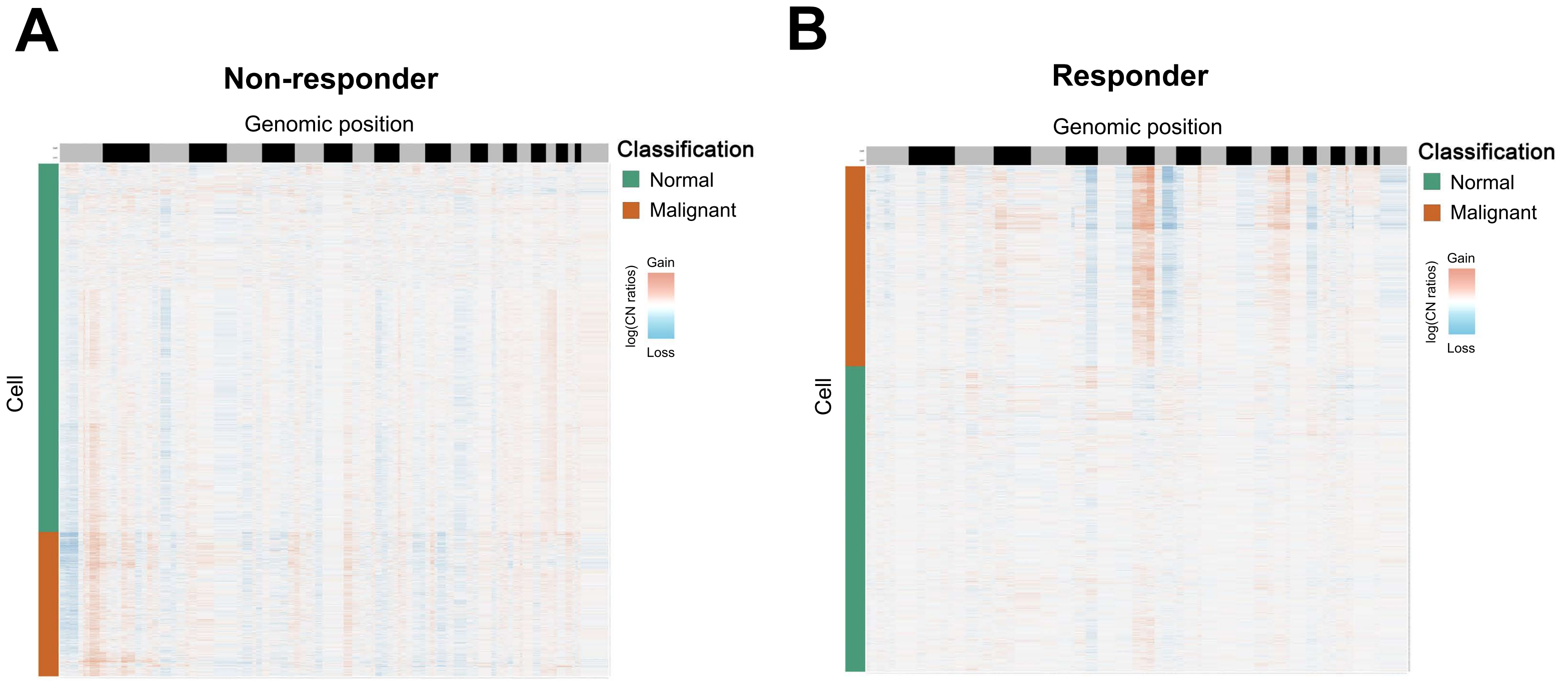

Supplemental Figure S9. Inference of single-nuclei malignant cells using copyKat to determine copy number alteration profiles

Heatmaps depict inferred large-scale chromosomal copy number alterations (CNAs) across the genome for individual single nuclei, in the Non-responder (A), and Responder (B). Each column represents a genomic position, while each row corresponds to a single nucleus. Gains (red) and losses (blue) in copy number are inferred relative to a diploid reference. The vertical annotation bars to the left of each heatmap denote cell classification by copyKat, where green indicates diploid (normal), and orange indicates aneuploid (malignant) cells. Top chromosomal track illustrates approximate genomic locations across autosomes.while the translation archetype expands significantly, accompanied by a moderate increase in the metabolism archetype.

Supplemental Document S1. Patient History

Pt 1 (non-responder)

The patient was a 68-year-old who had received multiple prior lines of therapies for recurrent stage IIIC low-grade serous ovarian carcinoma, metastatic to the liver and pleura. Treatment course over 8 years described below.

- Initial diagnosis: postmenopausal woman with GI symptoms and elevated CA 125 to 244.
    - optimal debulking surgery with evidence of stage IIIc low-grade ovarian carcinoma.
- 1st Line: IV/IP chemotherapy with **IP cisplatin plus IV/IP Taxol ×6**
- 2nd Line: recurrence 2 years after initial diagnosis via PET/CT in pelvis
    - Secondary debulking surgery for low-grade serous ovarian cancer.
    - Adjuvant chemotherapy with 3 cycles of **Doxil;** discontinued due to low ejection fraction of 40%, which resolved with discontinuation.
- 3rd line: **tamoxifen** 20 mg daily
- Abdominal hernia surgery which also demonstrated recurrent disease.
- 4th line: c**arboplatin plus Gemzar**. Partial response.
- 5th line: Palliative radiation to the occipital bony area with temporary improvement in her neck pain.
- 6th line: **niraparib;** with subsequent progression.
- 7th line: **carboplatin plus Taxotere x** 6 cycles
- 8th: **Letrozole.**
- Restaging CT scan with progression in the liver
- 9th line: **sirtex therapy for liver metastases**
- 10th liine: **Topotecan/bevacizumab**. Toxicity: discontinuation due to dental abscess.
- percutaneous **ablation of inferior right hepatic lobe for liver metastases**
- 11th line: **trametinib** (dose reduced to 1.5mg) with oligo progression
- 12th line: brachytherapy for aortocaval metastases
- 13th line: **weekly Nab-paclitaxel** x 6 cycles
- Treated again with targeted **yttrium-90 to liver metastases**
- 14th line: PIPAC with IP cisplatin/doxorubicin x 2 on clinical trial

PIPAC treatment on trial:

- PIPAC treatments #1 and #2 uncomplicated. No severe adverse events (SAEs.
- At PIPAC #2, a significant peritoneal response with PCI decreased from 20 at PIPAC #1 to 14 at PIPAC #2. The patient did not undergo PIPAC #3 as she was found to have disease progression in the chest cavity on imaging performed after PIPAC #2.
- CT CAP c/w systemic progression: Lung/pleura; heart (myocardium); liver parenchymal versus serosal metastases
- 15th line: single agent Abraxane. mixed response
- 16th line: Abraxane/Gemzar, complicated by severe bone marrow toxicity

- 17th line: clinical trial: A phase I study with an Oral TrkA Inhibitor VMD-928 for Treatment of Advanced Adult Solid Tumors

Biomarkers (HopeSeq): AKT3 amplification, MDM4 amplification, NTRK1 amplification, NRAS (c.182A>G, p.Q61R), MSI stable, CPS<1, CPS 0
Next generation sequencing of the tumor (Foundation one): NRAS and BLM mutations. BRCA 1/BRCA2 was non-mutated.

<u>Pt#2 (responder)</u>
59-year-old female with recurrent low-grade serous ovarian carcinoma and multiple prior lines of therapies. At presentation, patient had stage II borderline tumor, and over several years, subsequently developed stage IV invasive low grader serous ovarian cancer.
Prior Oncologic Treatment: Tamoxifen, carboplatin and paclitaxel, rucabarib, anastrozole.
Treatment course description over a course of 8 years below:

- Initial Diagnosis: Ovarian cancer.
  Initial primary surgery diagnosis of stage IIC serous borderline tumor, with a noninvasive implant on the sigmoid colon. Her pre-treatment CA-125 was 5500. No adjuvant therapy.
- Recurrence 4 years later: recurrent low-grade serous carcinoma diagnosis.
    - Surgery. Path: Low-grade serous carcinoma involving mesenteric and periappendiceal fat.
    - Initiated postoperative.
- 1st line: tamoxifen. Progression 3 months later with peritoneal metastases.
- 2nd line: **carboplatin/paclitaxel.** For peritoneal metastases
- Failed tertiary debulking attempt, with low-grade serous carcinoma involving small intestine tumor. She had unresectable disease.
- 3rd line: **Rucaparib**
- 4th line: **Opdivo** x 3 with GI toxicity
- Development of ascites requiring Paracenteses, hospitalizions for dehydration.
- 5th line: experimental therapy in Arizona with low dose chemotherapy twice weekly, with multiple agents that included:
  Carboplatin 20 mg
  Cytoxan 100 mg with mesna 100 mg
  Doxorubicin 10 mg
  Gemcitabine 160 mg
  Neville been 5 mg
  Taxotere 15 mg
- Vit C infusion + other vitamins
- Paracentesis
- 6th line: **Anastrozole**
- 7th line: PIPAC with IP cisplatin/doxorubicin
  PIPAC trial treatment:
    - Presented with progressive, abdominopelvic peritoneal, mesenteric, and serosal carcinomatosis with moderate abdominopelvic ascites.

    - enrolled on the PIPAC trial and underwent 6 cycles of PIPAC with IP cisplatin and doxorubicin.
    - Restaging CT scan after PIPAC #2 demonstrated a subtotal resolution of ascites and moderate decrease in serosal and peritoneal carcinomatosis. Additional partial response was noted on the laparoscopic assessment performed during PIPAC #3 with significant flattening of peritoneal and diaphragmatic nodules and regular appearance in the small bowel and mesenteric nodules.
- Underwent 6 cycles of PIPAC with near normalization of CA125, and resolution of ascites. Tolerated well. No SAEs. Completed therapy of 6 cycles and transitioned to next line of therapy.
- 8th line: trametinib with further reduction in CA125 and partial response on CT.

Biomarkers: By history, germline testing was negative.
Next generation sequencing of tumor (Foundation one): KMT2C (MLL3) mutation, MSI stable, tumor burden low; Combined Positive Score (CPS): 0, PD-L1 No Expression (CPS < 1)

Supplemental Document S2. Eligibility Criteria

Inclusion Criteria:

- Documented informed consent of the participant and/or legally authorized representative
- Patients must have histologically confirmed ovarian, uterine, gastric, appendiceal or colorectal cancer with peritoneal carcinomatosis
- Prior IP chemotherapy is permitted
- ECOG performance status ≤ 2
- Absolute neutrophil count (ANC) ≥ 1500/mm^3
- Platelets ≥ 100,000/mm^3
- Hemoglobin ≥ 9g/dL
- Serum total bilirubin ≤ 1.5x upper limit of normal (ULN)
- Alanine aminotransferase (ALT), serum glutamate pyruvate transaminase (SGPT), and aspartate aminotransferase (AST), serum glutamic oxaloacetic transaminase (SGOT) ≤ 2.5x ULN, unless liver metastases are present or unless patient is known to have chronic liver disease (hepatitis) in which case AST and ALT must be ≤ 5x ULN
- Alkaline phosphatase ≤ 2x ULN
- Serum creatinine ≤ 1.5x ULN, or creatinine clearance ≥ 40 mL/min as calculated by the Cockcroft-Gault formula
- No contraindications for a laparoscopy
- The peritoneal disease does not have to be measurable by RECIST 1.1 but needs to be visible on cross sectional imaging or diagnostic laparoscopy
- Patients must have progressed on at least one evidence-based chemotherapeutic regimen
- For patients with a known history of chronic hepatitis B virus (HBV) infection, the HBV viral load must be undetectable on suppressive therapy, if indicated
- Patients with a known history of hepatitis C virus (HCV) infection must have been treated and cured. For patients with HCV infection who are currently on treatment, they are eligible if they have an undetectable HCV viral load
- Women of childbearing potential (WOCBP) and male patients with WOCBP partner must be using an adequate method of contraception to avoid pregnancy throughout the study and for up to 12 weeks after the last dose of investigational product in such a manner that the risk of pregnancy is minimized. WOCBP include any female who has experienced menarche and who has not undergone successful surgical sterilization (hysterectomy, bilateral tubal ligation, or bilateral oophorectomy) or is not postmenopausal. Post menopause is defined as:
    - Amenorrhea ≥ 12 consecutive months without another cause or
    - For women with irregular menstrual periods and on hormone replacement therapy (HRT), a documented serum follicle stimulating hormone (FSH) level > 35 mIU/mL
    - Women who are using oral contraceptives, other hormonal contraceptives (vaginal products, skin patches, or implanted or injectable products), or mechanical products such as an intrauterine device or barrier methods (diaphragm, condoms, spermicides)

to prevent pregnancy, or are practicing abstinence or where their partner is sterile (e.g., vasectomy) should be considered to be of childbearing potential<br>• INCLUSION TO PROCEED WITH PIPAC: Laparoscopy findings must meet all of the criteria below in order to proceed to PIPAC:<br>o PIPAC access is feasible<br>o There is room for aerosol therapy<br>o There is no evidence of impending bowel obstruction<br>o ≤ 5L of ascites<br>o Not a candidate for cytoreduction and HIPEC
Exclusion Criteria:<br><br>• Previous treatment with maximum cumulative doses of doxorubicin, daunorubicin, epirubicin, idarubicin, and/or other anthracyclines and anthracenediones

Table S1 - Sample information - Non-responder

Cycle	Sample	Quadrant	Pre Post	Normal/Tumor	Sequencing	Tissue Type	Pathology	Pathology label
C1-Pre	A	RLQ	Pre	Normal	sequenced	RLQ bx pre pipac normal	Fibroadipose tissue, negative for carcinoma	Negative
C1-Pre	B	RLQ	Pre	Tumor	sequenced	RLQ bx pre pipac tumor	Recurrent low-grade serous carcinoma	Positive
C1-Pre	C	LLQ	Pre	Tumor	sequenced	LLQ bx pre pipac tumor	Recurrent low-grade serous carcinoma	Positive
C2-Pre	D	LUQ	Pre	Tumor		LUQ bx pre pipac tumor	Metastatic low-grade serous carcinoma	Positive
C2-Pre	E	RLQ	Pre	Tumor	sequenced	RLQ bx pre pipac tumor	Metastatic low-grade serous carcinoma	Positive
C2-Pre	F	RLQ	Pre	Tumor	sequenced	RLQ bx pre pipac tumor	Metastatic low-grade serous carcinoma	Positive
C2-Pre	G	RUQ	Pre	Tumor		RUQ bx pre pipac tumor	Metastatic low-grade serous carcinoma	Positive
C2-Pre	H	LLQ	Pre	Tumor	sequenced	LLQ bx pre pipac tumor	Metastatic low-grade serous carcinoma	Positive
C2-Pre	I	RUQ	Pre	Normal		RUQ bx pre pipac normal	Fat necrosis, negative for tumor	Negative
C2-Post	J	RLQ	Post	Tumor	sequenced	RLQ bx post pipac tumor	Metastatic low-grade serous carcinoma	Positive
C2-Post	K	RLQ	Post	Normal	sequenced	RLQ bx post pipac normal	Metastatic low-grade serous carcinoma	Positive

Supplementary Table S1. Sample information - Responder

Cycle	Sample	Quadrant	Pre Post	Normal/Tumor	Sequencing	tissue type	Pathology	Pathology label
C1-Pre	A	RLQ	Pre	Tumor	Sequence	RLQ bx pre pipac tumor	Low grade serous carcinoma	Positive
C1-Post	B	RLQ	Post	Tumor		RLQ bx post pipac tumor	Low grade serous carcinoma	Positive
C1-Pre	C	RUQ	Pre	Tumor	Sequence	RUQ bx pre pipac tumor	Low grade serous carcinoma	Positive
C1-Pre	D	LLQ	Pre	Tumor	Sequence	LLQ bx pre pipac tumor	Low grade serous carcinoma	Positive
C1-Pre	E	LUQ	Pre	Normal		LUQ bx pre pipac normal	Low grade serous carcinoma	Positive
C1-Post	F	RLQ	Post	Normal		RLQ bx post pipac normal	Low grade serous carcinoma	Positive
C1-Pre	G	Ascites	Pre	Pipac		ascites pre pipac		
C2-Pre	H	RLQ	Pre	Tumor		RLQ bx pre pipac tumor	Involved by invasive low grade serous carcinoma	Positive
C2-Post	I	Left Flank	Post	Tumor		left flank bx post pipac tumor	Involved by invasive low grade serous carcinoma	Positive
C2-Pre	J	LUQ	Pre	Tumor	Sequence	LUQ bx pre pipac tumor	Involved by invasive low grade serous carcinoma	Positive
C2-Pre	K	RUQ	Pre	Tumor	Sequence	RUQ bx pre pipac tumor	Involved by invasive low grade serous carcinoma	Positive
C2-Pre	L	LLQ	Pre	Tumor	Sequence	LLQ bx pre pipac tumor	Involved by invasive low grade serous carcinoma	Positive
C2-Pre	N	Left Flank	Pre	Normal	Sequence - failed QC	left flank bx pre pipac normal	Benign fibrous tissue, negative for tumor	Negative
C2-Post	O	Left Flank	Post	Normal		left flank bx post pipac normal	Involved by invasive low grade serous carcinoma	Positive
C2-Unk	P	Wash	Unk	Washings		pelvic washings		
C3-Pre	Q	RLQ	Pre	Tumor		RLQ bx pre pipac tumor	Viable low grade serous carcinoma	Positive
C3-Post	R	RLQ	Post	Tumor		RLQ bx post pipac tumor	Viable low grade serous carcinoma	Positive
C3-Pre	S	LUQ	Pre	Tumor	Sequence - failed QC	LuQ bx pre pipac tumor	Viable low grade serous carcinoma	Positive
C3-Pre	T	RUQ	Pre	Tumor	Sequence	RuQ bx pre pipac tumor	Viable low grade serous carcinoma	Positive
C3-Pre	U	RLQ	Pre	Normal		RLQ bx pre pipac normal	Pathology missing	
C3-Pre	V	Right Flank	Pre	Normal		R Flank bx pre pipac normal	Dense fibrous tissue, negative for carcinoma	Negative
C3-Post	W	RLQ	Post	Tumor		pelvis tumor post pipac	No viable carcinoma identified	Negative
C4-Pre	X	RLQ	Pre	Tumor	Sequence	RLQ bx pre pipac tumor	Low-grade serous carcinoma, associated with calcifications	Positive
C4-Post	Y	RLQ	Post	Tumor		RLQ bx post pipac tumor	Low-grade serous carcinoma, associated with calcifications	Positive
C4-Pre	Z	RUQ	Pre	Tumor	Sequence	RUQ bx pre pipac tumor	Low-grade serous carcinoma, associated with calcifications	Positive
C4-Pre	AA	LUQ	Pre	Tumor	Sequence	LUQ bx pre pipac tumor	Connective tissue fragment; negative for carcinoma	Negative
C4-Pre	AB	RUQ	Pre	Normal		RUQ bx pre pipac normal	Connective tissue fragment; negative for carcinoma	Negative
C4-Post	AC	RLQ	Post	Normal		RLQ bx post pipac normal	Connective tissue fragment; negative for carcinoma	Negative
C4-Unk	AD	Wash	Unk	Washing		peritoneal washing		
C5-Pre	AE	RUQ	Pre	Tumor	Sequence	RUQ bx pre pipac tumor	Low-grade serous carcinoma, associated with calcifications	Positive
C5-Post	AF	RUQ	Post	Tumor		RUQ bx post pipac tumor	Low-grade serous carcinoma, associated with calcifications	Positive
C5-Pre	AG	LUQ	Pre	Tumor		LUQ bx pre pipac tumor	Mesothelial-lined connective tissue; negative for carcinoma	Negative
C5-Pre	AH	LUQ	Pre	Normal		LUQ bx pre pipac normal	Connective tissue fragment; negative for carcinoma	Negative
C5-Pre	AI	Wash	Pre	Washing		pre pelvic wash		
C6-Pre	AJ	LLQ	Pre	Tumor	Sequence	LLQ bx pre pipac tumor	[illegible] low grade serous carcinoma with psammomatous calcifications	Positive
C6-Post	AK	LUQ	Post	Tumor		LUQ bx post pipac tumor	Fragments of benigin connective tissue; no carcinoma identified	Negative
C6-Pre	AL	LUQ	Pre	Tumor		LUQ bx pre pipac tumor	Fragments of benigin skeletal muscles; no carcinoma identified	Negative
C6-Pre	AM	R	Pre	Tumor	Sequence	R flank pre pipac tumor	[illegible] low grade serous carcinoma with psammomatous calcifications	Positive
C6-Pre	AN	R	Pre	Normal	Sequence	R flank pre pipac normal	Fragments of benigin connective tissue; no carcinoma identified	Negative
C6-Pre	AO	LUQ	Pre	Normal		LUQ pre pipac normal	[illegible]ctive tissue with scattered calcifications; no carcinoma identified	Negative
C6-Post	AP	LUQ	Post	Normal		LUQ bx post pipac normal	Fragments of benigin connective tissue; no carcinoma identified	Negative

Table S2 - Cancer Cell Pathways Changing Over Treatment Cycles (Linear Modeling) - Non-responder

**Pathway**	**Estimate**	**p value**	**FDR**	**Significance (FDR < 0.05)**
HALLMARK-ADIPOGENESIS	-0.0018977	0.23872127	0.30605291	Not Significant
HALLMARK-ALLOGRAFT-REJECTION	0.00863053	0.00031544	0.00063089	Significant
HALLMARK-ANDROGEN-RESPONSE	-0.0099301	0.00116991	0.00208913	Significant
HALLMARK-ANGIOGENESIS	0.03427628	6.84E-15	3.80E-14	Significant
HALLMARK-APICAL-JUNCTION	0.00637815	0.00222591	0.00383778	Significant
HALLMARK-APICAL-SURFACE	-0.0009903	0.81969616	0.81969616	Not Significant
HALLMARK-APOPTOSIS	0.01355509	4.69E-10	1.67E-09	Significant
HALLMARK-BILE-ACID-METABOLISM	-0.0007153	0.70628531	0.75136735	Not Significant
HALLMARK-CHOLESTEROL-HOMEOSTASIS	0.00230125	0.37139261	0.43185188	Not Significant
HALLMARK-COAGULATION	0.01449603	5.47E-10	1.82E-09	Significant
HALLMARK-COMPLEMENT	0.00358098	0.06461045	0.08973673	Not Significant
HALLMARK-DNA-REPAIR	0.01171609	7.27E-10	2.27E-09	Significant
HALLMARK-E2F-TARGETS	0.02281725	1.41E-18	1.00E-17	Significant
HALLMARK-EPITHELIAL-MESENCHYMAL-TRANSITION	0.02151786	1.10E-18	9.18E-18	Significant
HALLMARK-ESTROGEN-RESPONSE-EARLY	-0.0369526	6.71E-81	3.35E-79	Significant
HALLMARK-ESTROGEN-RESPONSE-LATE	-0.0176859	4.66E-21	5.83E-20	Significant
HALLMARK-FATTY-ACID-METABOLISM	-0.0089165	8.04E-08	2.01E-07	Significant
HALLMARK-G2M-CHECKPOINT	0.02466726	5.24E-23	8.74E-22	Significant
HALLMARK-GLYCOLYSIS	0.00057886	0.73640992	0.75790303	Not Significant
HALLMARK-HEDGEHOG-SIGNALING	-0.0124463	0.01540503	0.02407036	Significant
HALLMARK-HEME-METABOLISM	-0.0012504	0.47086901	0.53507842	Not Significant
HALLMARK-HYPOXIA	-0.0151375	2.91E-15	1.82E-14	Significant
HALLMARK-IL2-STAT5-SIGNALING	-0.0122425	9.79E-11	3.76E-10	Significant
HALLMARK-IL6-JAK-STAT3-SIGNALING	-0.0180073	5.78E-09	1.61E-08	Significant
HALLMARK-INFLAMMATORY-RESPONSE	-0.0029909	0.14828186	0.20038089	Not Significant
HALLMARK-INTERFERON-ALPHA-RESPONSE	0.00574533	0.03230026	0.04614323	Significant
HALLMARK-INTERFERON-GAMMA-RESPONSE	0.0046413	0.02593864	0.03814506	Significant
HALLMARK-KRAS-SIGNALING-DN	-0.0074398	0.00010695	0.00022282	Significant
HALLMARK-KRAS-SIGNALING-UP	0.01378627	3.97E-13	1.99E-12	Significant
HALLMARK-MITOTIC-SPINDLE	0.00998913	2.80E-05	6.36E-05	Significant
HALLMARK-MTORC1-SIGNALING	0.01122115	1.61E-09	4.74E-09	Significant
HALLMARK-MYC-TARGETS-V1	0.03449531	8.62E-27	2.16E-25	Significant
HALLMARK-MYC-TARGETS-V2	0.0143941	7.31E-09	1.92E-08	Significant
HALLMARK-MYOGENESIS	-0.0024354	0.18357249	0.24154275	Not Significant
HALLMARK-NOTCH-SIGNALING	-0.0285997	4.57E-11	1.90E-10	Significant
HALLMARK-OXIDATIVE-PHOSPHORYLATION	0.00674745	9.00E-05	0.00019558	Significant
HALLMARK-P53-PATHWAY	0.00186101	0.2982387	0.37279837	Not Significant
HALLMARK-PANCREAS-BETA-CELLS	0.00288662	0.58418063	0.64908959	Not Significant
HALLMARK-PEROXISOME	0.00103547	0.62668118	0.6811752	Not Significant
HALLMARK-PI3K-AKT-MTOR-SIGNALING	0.00329073	0.32683315	0.39857702	Not Significant
HALLMARK-PROTEIN-SECRETION	0.0090927	0.00794868	0.01282045	Significant
HALLMARK-REACTIVE-OXYGEN-SPECIES-PATHWAY	-0.0159205	6.34E-06	1.51E-05	Significant
HALLMARK-SPERMATOGENESIS	-0.0082013	0.00053367	0.00098828	Significant
HALLMARK-TGF-BETA-SIGNALING	-0.0132694	0.00400863	0.00668105	Significant
HALLMARK-TNFA-SIGNALING-VIA-NFKB	-0.0078981	0.0004687	0.00090134	Significant
HALLMARK-UNFOLDED-PROTEIN-RESPONSE	0.01995509	3.11E-19	3.11E-18	Significant
HALLMARK-UV-RESPONSE-DN	-0.0187713	2.04E-11	9.26E-11	Significant
HALLMARK-UV-RESPONSE-UP	-0.0015782	0.35993485	0.42849387	Not Significant
HALLMARK-WNT-BETA-CATENIN-SIGNALING	0.00114648	0.74274497	0.75790303	Not Significant
HALLMARK-XENOBIOTIC-METABOLISM	0.00349218	0.01776467	0.02691616	Significant

Table S2 - Cancer Cell Pathways Changing Over Treatment Cycles (Linear Modeling) - Responder

Pathway	Estimate	p value	FDR	Significance (FDR < 0.05)
HALLMARK-ADIPOGENESIS	-9.50E-05	0.84426082	0.84426082	Not Significant
HALLMARK-ALLOGRAFT-REJECTION	-0.0032856	2.65E-06	7.80E-06	Significant
HALLMARK-ANDROGEN-RESPONSE	-0.0041703	8.14E-05	0.00016275	Significant
HALLMARK-ANGIOGENESIS	0.00265082	0.03383456	0.04572238	Significant
HALLMARK-APICAL-JUNCTION	0.00250822	0.00054135	0.0009667	Significant
HALLMARK-APICAL-SURFACE	0.0088674	7.16E-14	4.47E-13	Significant
HALLMARK-APOPTOSIS	-0.0010375	0.07375135	0.09218918	Not Significant
HALLMARK-BILE-ACID-METABOLISM	0.00091391	0.0721065	0.09218918	Not Significant
HALLMARK-CHOLESTEROL-HOMEOSTASIS	0.00202795	0.00217026	0.0036171	Significant
HALLMARK-COAGULATION	-0.00021	0.74819982	0.7634692	Not Significant
HALLMARK-COMPLEMENT	0.00042212	0.37834748	0.41124726	Not Significant
HALLMARK-DNA-REPAIR	0.00046439	0.29527301	0.33553751	Not Significant
HALLMARK-E2F-TARGETS	-0.007319	4.02E-43	1.01E-41	Significant
HALLMARK-EPITHELIAL-MESENCHYMAL-TRANSITION	-0.0036892	4.31E-06	1.13E-05	Significant
HALLMARK-ESTROGEN-RESPONSE-EARLY	0.0025474	5.93E-08	2.47E-07	Significant
HALLMARK-ESTROGEN-RESPONSE-LATE	-0.0011986	0.01699456	0.02427794	Significant
HALLMARK-FATTY-ACID-METABOLISM	0.00070013	0.12531052	0.15281771	Not Significant
HALLMARK-G2M-CHECKPOINT	-0.0058805	4.61E-24	7.68E-23	Significant
HALLMARK-GLYCOLYSIS	0.0011075	0.01229223	0.01807681	Significant
HALLMARK-HEDGEHOG-SIGNALING	-0.0027229	0.06634801	0.08730001	Not Significant
HALLMARK-HEME-METABOLISM	0.00226839	1.48E-06	4.63E-06	Significant
HALLMARK-HYPOXIA	0.00239051	3.57E-06	9.90E-06	Significant
HALLMARK-IL2-STAT5-SIGNALING	0.0026445	2.74E-07	1.05E-06	Significant
HALLMARK-IL6-JAK-STAT3-SIGNALING	0.00243506	0.00352481	0.00568517	Significant
HALLMARK-INFLAMMATORY-RESPONSE	0.00265994	2.65E-05	6.11E-05	Significant
HALLMARK-INTERFERON-ALPHA-RESPONSE	-0.0020664	0.00656989	0.00995438	Significant
HALLMARK-INTERFERON-GAMMA-RESPONSE	-0.0026988	4.69E-06	1.17E-05	Significant
HALLMARK-KRAS-SIGNALING-DN	0.0055169	7.26E-24	9.08E-23	Significant
HALLMARK-KRAS-SIGNALING-UP	0.00038963	0.46616114	0.4959161	Significant
HALLMARK-MITOTIC-SPINDLE	-0.0036423	3.28E-05	7.12E-05	Significant
HALLMARK-MTORC1-SIGNALING	-0.0018803	2.69E-05	6.11E-05	Significant
HALLMARK-MYC-TARGETS-V1	-0.0094904	8.58E-44	4.29E-42	Significant
HALLMARK-MYC-TARGETS-V2	0.00090116	0.20451921	0.24347525	Not Significant
HALLMARK-MYOGENESIS	0.00333315	4.73E-07	1.69E-06	Significant
HALLMARK-NOTCH-SIGNALING	0.00817174	6.17E-11	3.08E-10	Significant
HALLMARK-OXIDATIVE-PHOSPHORYLATION	-0.0023057	8.94E-07	2.98E-06	Significant
HALLMARK-P53-PATHWAY	0.00124389	0.00537822	0.00840346	Significant
HALLMARK-PANCREAS-BETA-CELLS	0.00546073	0.00017945	0.0003451	Significant
HALLMARK-PEROXISOME	0.00052453	0.34402446	0.3822494	Not Significant
HALLMARK-PI3K-AKT-MTOR-SIGNALING	0.00619021	1.91E-08	8.68E-08	Significant
HALLMARK-PROTEIN-SECRETION	-0.0042258	6.35E-05	0.00013233	Significant
HALLMARK-REACTIVE-OXYGEN-SPECIES-PATHWAY	-0.0028452	0.00122619	0.00211412	Significant
HALLMARK-SPERMATOGENESIS	0.00067024	0.29338505	0.33553751	Not Significant
HALLMARK-TGF-BETA-SIGNALING	0.00572593	0.00032801	0.00060743	Significant
HALLMARK-TNFA-SIGNALING-VIA-NFKB	0.00496072	3.77E-19	3.77E-18	Significant
HALLMARK-UNFOLDED-PROTEIN-RESPONSE	-0.0012539	0.02950652	0.04098127	Significant
HALLMARK-UV-RESPONSE-DN	0.00056738	0.56900619	0.59271478	Not Significant
HALLMARK-UV-RESPONSE-UP	0.00299316	5.94E-11	3.08E-10	Significant
HALLMARK-WNT-BETA-CATENIN-SIGNALING	0.00774614	4.60E-16	3.28E-15	Significant
HALLMARK-XENOBIOTIC-METABOLISM	0.00354319	1.77E-18	1.47E-17	Significant

Table S3 - Cancer Cell Genes Changing Over Treatment Cycles (Linear Modeling)

Available at: https://doi.org/10.6084/m9.figshare.30279679.v1

Table S4 - Immunostaining of pre-treatment sample from responder and non-responder

Slide name	Patient	Cycle	Quadrant	density_t	density_cd8t	density_cd4t	density_cd4treg	density_mac	density_fibro	percent_of_t	percent_of_cd8t	percent_of_cd4t	percent_of_cd4treg	percent_of_mac	percent_of_fibro	percent_cd4_of_cd3	percent_treg_of_cd4
Nonresponder 1 cycle RLQ_Scan1_Stitched.ome.tif	Nonresponder	1	RLQ	267.2751485	65.52808248	201.747066	60.5638338	459.4908571	647.7351668	4.319086125	1.058914132	3.260171993	0.978693364	7.425234245	10.46720575	75.48291233	30.01968504
Nonresponder 2 cycle RLQ_Scan1_Stitched.ome.tif	Nonresponder	2	RLQ	283.4017579	68.61983491	214.7819229	66.96412844	362.5997175	1152.831623	7.648197796	1.851851852	5.796345944	1.807169099	9.785522788	31.11160759	75.78708212	31.17773019
Nonresponder 2 cycle RLQpre c_Scan1_Stitched.ome.tif	Nonresponder	2	RLQ	273.8696398	30.07720267	243.7924372	81.37554277	733.3824583	1217.29123	0.866926745	7.026922892	2.34551847	21.13856379	35.08645186	35.08645186	89.01769372	33.37902673
Nonresponder 1 cycle RLQpre A - DONE_Scan1_Stitched.ome.tif	Nonresponder	1	RLQ	380.9833753	44.34109727	336.642278	99.18403338	707.5127714	2555.058491	0.637530409	4.84019797	1.426054861	10.17252468	36.7362917	36.7362917	88.36140888	29.4627383
Nonresponder 2 cycle LLQpre E_Scan1_Stitched.ome.tif	Nonresponder	2	LLQ	213.3732524	37.82731757	175.5459348	92.8694623	943.8708522	2486.636481	0.7848482	3.642259611	1.926872826	19.58360748	51.59319485	51.59319485	82.27176221	52.90322581
Nonresponder 1 cycle LLQpre D_Scan3_Stitched.ome.tif	Nonresponder	1	LLQ	256.0409211	46.11316338	209.9277578	65.78381349	1095.751952	3643.907316	0.620202108	2.823437568	0.884763846	14.73738995	49.00897775	49.00897775	81.98992443	31.33640553
Responder 1 cycle RLQ_Scan1_Stitched.ome.tif	Responder	1	RLQ	173.2509049	64.82642553	108.4244793	4.108717111	296.5124182	989.7442997	4.903734333	1.834862385	3.068871947	0.116294095	8.392557178	28.01395529	62.58234519	3.789473684
Responder 2 cycle LLQ_Scan1_Stitched.ome.tif	Responder	2	LLQ	110.7039547	26.61152758	84.09242716	5.322305516	336.3697086	514.8443536	2.681564246	0.64460679	2.036957456	0.128921358	8.147829824	12.47099269	75.96153846	6.329113924
Responder 2 cycle RUQ_Scan1_Stitched.ome.tif	Responder	2	RUQ	169.01291	108.5102341	60.50267596	5.261102257	135.4733831	352.4938513	1.923076923	1.234660281	0.688416642	0.059862317	1.541454654	4.010775217	35.79766537	8.695652174
Responder 3 cycle LUQ_Scan1_Stitched.ome.tif	Responder	3	LUQ	384.7327184	188.1844818	196.5482366	1.045469344	325.1409659	721.3738471	6.523666017	3.190923595	3.332742422	0.017727353	5.513206878	12.23187378	51.08695652	0.531914894
Responder 3 cycle RLQ_Scan1_Stitched.ome.tif	Responder	3	RLQ	366.5696941	11.78742822	354.7822659	42.51203622	217.0046213	716.7142834	9.934017595	0.319438626	9.614578969	1.152073733	5.880812736	19.42291579	96.78439642	11.98257081
Responder 3 cycle RUQ_Scan1_Stitched.ome.tif	Responder	3	RUQ	310.5880738	1.773522192	308.8145516	36.35720493	96.43526917	1219.518197	8.488851188	0.048473097	8.44037809	0.993698497	2.635724673	33.33131362	99.4289793	11.77315147
Responder 4 cycle LUQ_Scan1_Stitched.ome.tif	Responder	4	LUQ	788.1033508	348.2229772	439.8803736	40.19402218	46.57998832	551.0713134	13.31049359	5.881233346	7.429260246	0.678847862	0.786702195	9.307194518	55.81506196	9.137489325
Responder 4 cycle RLQ#2_Scan1_Stitched.ome.tif	Responder	4	RLQ	276.418339	13.62356622	262.7947728	10.63147458	395.0197589	945.6919451	5.341764677	0.263274445	5.078490232	0.205452488	7.633728655	18.27542936	95.07139567	4.045542636
Responder 4 cycle RLQ_Scan1_Stitched.ome.tif	Responder	4	RLQ	627.6164791	290.3259903	337.2904888	9.758856818	288.4962047	226.2834925	8.795623558	4.068723823	4.726899735	0.136763826	4.043080605	3.171211215	53.7414966	2.893309222
Responder 5 cycle RUQ_Scan1_Stitched.ome.tif	Responder	5	RUQ	1180.741727	430.3836504	750.3580762	55.20461229	264.5574881	801.9531562	20.48854174	7.468130573	13.02041117	0.957924987	4.590671284	13.9157026	63.54972127	7.357102434
Responder 6 cycle LLQ_Scan1_Stitched.ome.tif	Responder	6	LLQ	107.516609	51.7103691	55.80623992	0.767975779	176.6344291	2682.795387	2.003147804	0.963418706	1.039729098	0.014308199	3.290885677	49.9833071	51.9047619	1.376146789
Responder 1 cycle RUQpre_Stitched.ome.tif	Responder	1	RUQ	266.8025803	139.2375966	127.5649837	3.335032254	150.0764514	2106.906627	3.054691787	2.798609841	0.07316627	3.292482166	46.22279129	46.22279129	47.8125	2.614379085
